# Colorectal Cancer: Pathogenesis and Targeted Therapy

**DOI:** 10.1002/mco2.70127

**Published:** 2025-03-06

**Authors:** Jingyuan Li, Jiashu Pan, Lisheng Wang, Guang Ji, Yanqi Dang

**Affiliations:** ^1^ Institute of Digestive Diseases China‐Canada Center of Research for Digestive Diseases Longhua Hospital Shanghai University of Traditional Chinese Medicine Shanghai China; ^2^ State Key Laboratory of Integration and Innovation of Classic Formula and Modern Chinese Medicine (Shanghai University of Traditional Chinese Medicine) Shanghai China; ^3^ Department of Biochemistry Microbiology and Immunology Faculty of Medicine University of Ottawa Ottawa Ontario Canada; ^4^ China‐Canada Centre of Research for Digestive Diseases University of Ottawa Ottawa Ontario Canada

**Keywords:** colorectal cancer, genetics, epigenetics, tumor metabolism, molecular mechanisms, targeted therapy

## Abstract

Colorectal cancer (CRC) ranks among the most prevalent malignant neoplasms globally. A growing body of evidence underscores the pivotal roles of genetic alterations and dysregulated epigenetic modifications in the pathogenesis of CRC. In recent years, the reprogramming of tumor cell metabolism has been increasingly acknowledged as a hallmark of cancer. Substantial evidence suggests a crosstalk between tumor cell metabolic reprogramming and epigenetic modifications, highlighting a complex interplay between metabolism and the epigenetic genome that warrants further investigation. Biomarkers associated with the pathogenesis and metabolic characteristics of CRC hold significant clinical implications. Nevertheless, elucidating the genetic, epigenetic, and metabolic landscapes of CRC continues to pose considerable challenges. Here, we attempt to summarize the key genes driving the onset and progression of CRC and the related epigenetic regulators, clarify the roles of gene expression and signaling pathways in tumor metabolism regulation, and explore the potential crosstalk between epigenetic events and tumor metabolic reprogramming, providing a comprehensive mechanistic explanation for the malignant progression of CRC. Finally, by integrating reliable targets from genetics, epigenetics, and metabolic processes that hold promise for translation into clinical practice, we aim to offer more strategies to overcome the bottlenecks in CRC treatment.

## Graphical abstract

Chromosomal instability, microsatellite instability, and the CpG island methylator phenotype are key pathogenic pathways in the adenoma–carcinoma sequence of CRC. Epigenetic remodeling and metabolic reprogramming are two major hallmarks of tumor cells, demonstrating bidirectional regulatory mechanisms across various cancers, including CRC. Researchers are developing drugs that target critical elements within these pathogenic and metabolic pathways to enhance treatment strategies for colorectal cancer. *Abbreviations*: CIN, chromosomal instability; MSI, microsatellite instability; CIMP, CpG island methylator phenotype.

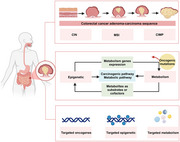

## 1 Introduction

Colorectal cancer (CRC) ranks as the third most prevalent malignancy globally and are the second leading cause of cancer‐related mortality [1]. In 2022, the number of patients succumbing to CRC constituted nearly one‐tenth of the total cancer‐related mortalities. Projections indicate that by 2040, the disease burden associated with CRC will surge by 63% [2]. Significantly, the incidence of CRC is demonstrating a tendency toward a younger age of onset, with the number of patients under 50 years old rising steadily year after year [3]. Although CRC screening has been shown to decrease the risk of CRC‐related mortality [4], participation and the associated benefits remain constrained by factors such as economic costs, patient awareness, and the potential for complications. Consequently, there exists a pressing clinical imperative to conduct a comprehensive investigation into the molecular mechanisms underpinning the development of CRC to identify potential biomarkers and therapeutic targets.

CRC exhibits considerable heterogeneity, influenced by both genetic and environmental factors that play a critical role in its pathogenesis. Recent advancements in genome sequencing have identified key driver genes involved in CRC progression, and their related oncogenic pathways have been extensively studied. The transition from normal colorectal epithelial cells to adenomatous lesions is largely driven by the accumulation of specific genetic alterations and the dysregulation of signaling pathways. The roles of chromosomal instability (CIN), microsatellite instability (MSI), the CpG island methylator phenotype (CIMP), and mutations within the adenoma–carcinoma sequence warrant further investigation [5]. Although the accumulation of genetic alterations is frequently regarded as the principal factor driving the malignant phenotype of CRC, recent studies have highlighted the regulatory role of epigenetic alterations in gene silencing and CRC progression. Epigenetic modification pertains to the regulation of gene expression and cellular function through alterations in chromatin structure that do not involve changes to the DNA sequence. These reversible modifications, which encompass DNA methylation, histone modification, and noncoding RNA (ncRNA) modification, are integral to the regulation of CRC biological processes [6]. In particular, DNA methylation has been the subject of extensive research, with genome‐wide hypomethylation and hypermethylation of CpG islands identified as key characteristics in the transition from precancerous to malignant states [7]. Growing evidence indicates a strong association between histone and ncRNA modifications and various malignant tumors, impacting cancer cell cycles, metabolism, and immune responses [8].

Additionally, metabolic reprogramming is a hallmark of CRC, characterized by tumor cells competing for nutrients to sustain their growth and survival within a dynamic microenvironment. This adaptive metabolic shift is regulated by oncogenes and tumor suppressor genes and is likely influenced by epigenetic modifications. The progression from sporadic benign adenomas to malignant CRC is characterized by alterations in glucose, lipid, and amino acid metabolism, thereby highlighting the investigation of metabolic reprogramming as a crucial area of focus in elucidating CRC pathogenesis [9]. Epigenetic remodeling and metabolic reprogramming demonstrate intricate bidirectional regulatory mechanisms across various cancers, including CRC. The aberrant accumulation of metabolites and the dysregulation of metabolic signals act as substrates and cofactors for epigenetic modifications, thereby influencing the expression of metabolic genes and enzymes. In the development of CRC, there is a significant intersection between epigenetic modifications and tumor metabolism. The interaction between the metabolome and the epigenome serves as a pivotal mechanism that regulates cellular states and tumor heterogeneity.

This review meticulously pinpoints the key oncogenes and epigenetic modifications during the transformation of normal colorectal epithelial cells into precancerous and malignant phenotypes. It places a particular emphasis on metabolic reprogramming, which safeguards the biological capabilities of tumor cells throughout the progression of CRC. To delve into the latent connection between epigenetic modifications and metabolic processes, this review zeroes in on metabolic pathways and typical metabolites, striving to uncover the underlying pathogenesis at a deeper level. Finally, it comprehensively summarizes the existing drugs targeting CRC. However, there are pressing practical issues that demand immediate resolution, such as the discovery of more potent targeted drugs and their expeditious translation into clinical applications (Graphic Abstract).

## 2 Molecular Mechanisms of CRC

**FIGURE 1:**
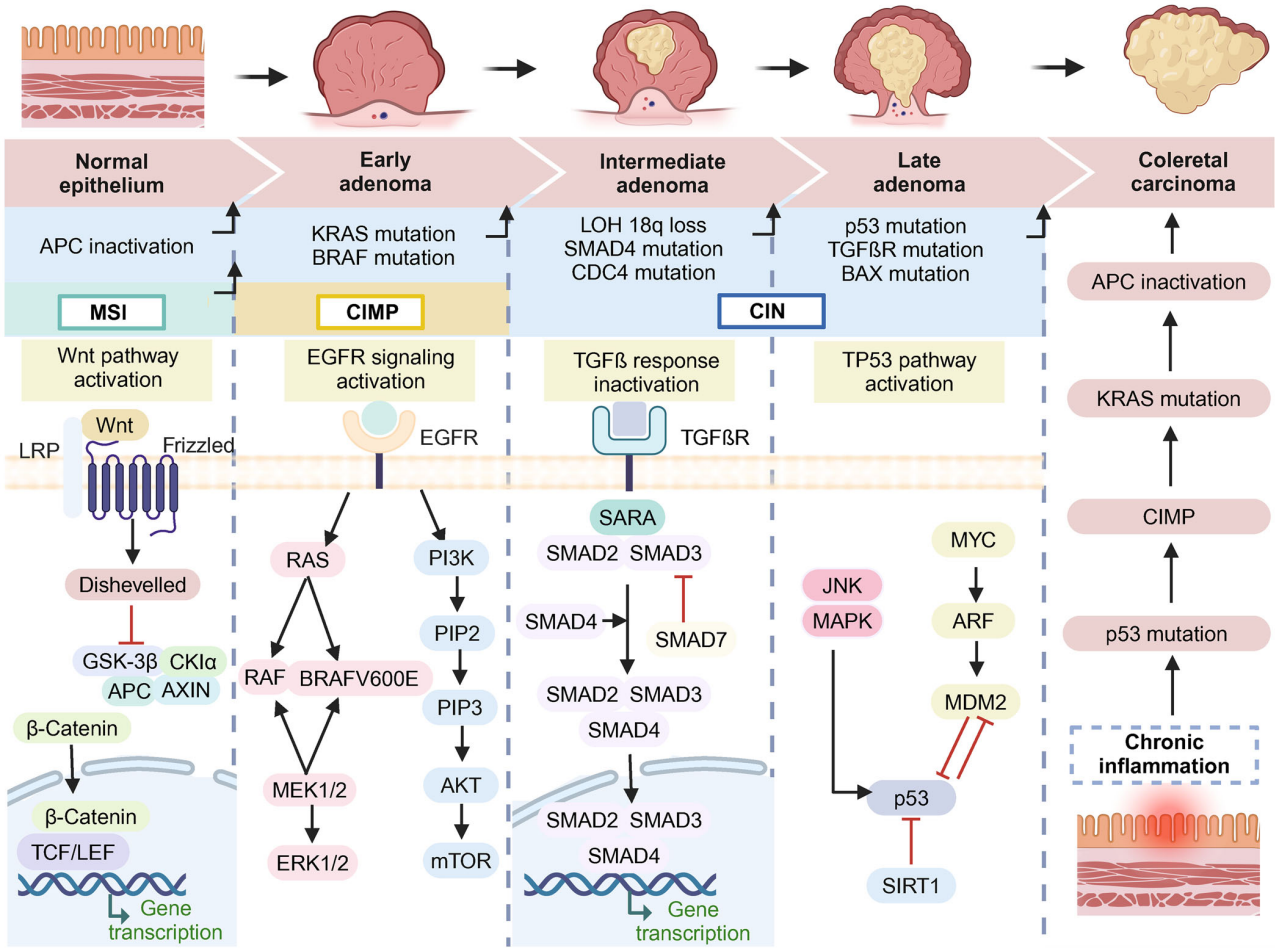
Molecular mechanisms related to the development of CRC. Mechanistic studies of CRC carcinogenesis primarily focus on CIN, MSI, and CIMP. Cancer progression involves the cumulative mutations of oncogenes and tumor suppressor genes, along with the activation of multiple signaling pathways. While there is a strong association between chronic inflammation and the development of CRC, the molecular events driving the disease and the adenoma–carcinoma sequence. *Abbreviations*: APC, adenomatous polyposis coli; KRAS, Kirsten rat sarcoma viral oncogene homolog; BRAF, B‐raf proto‐oncogene, serine/threonine kinase; LOH 18q, loss of heterozygosity on chromosome 18q; SMAD4, SMAD family member 4; CDC4, cell division cycle 4; p53, tumor protein p53; BAX, BCL2 associated X, apoptosis regulator; Wnt, wingless‐related integration site; LRP, low‐density lipoprotein receptor‐related protein; GSK‐3β, glycogen synthase kinase 3 beta; CKIα, cyclin‐dependent kinase inhibitor alpha; AXIN, Axin family proteins; TCF/LEF, T cell factor/lymphoid enhancer factor; EGFR, epidermal growth factor receptor; RAS, rat sarcoma; RAF, rapidly accelerated fibrosarcoma; BRAFV600E, BRAF 600 amino acid substitution mutation from V to E; MEK1/2, mitogen‐activated extracellular signal‐regulated kinase 1 and 2; ERK1/2, extracellular signal‐regulated kinase 1/2; PI3K, phosphatidylinositol 3‐kinase; PIP2, phosphatidylinositol 4,5‐bisphosphate; PIP3, phosphatidylinositol 3,4,5‐trisphosphate; AKT, protein kinase B; mTOR, mammalian target of rapamycin; TGFßR, transforming growth factor beta receptor; SARA, Smad anchor for receptor activation; JNK, c‐Jun N‐terminal kinases; MAPK, mitogen‐activated protein kinase; MYC, myelocytomatosis oncogene; ARF, alternative reading frame; MDM2, murine double minute 2; SIRT1, sirtuin 1.

The progression from normal colonic epithelium to CRC can occur through various pathways, primarily categorized into three major types: CIN, MSI, and CIMP. Each pathway is characterized by distinct genetic alterations, histopathological features, and clinical outcomes (Figure 1).

### 2.1 Chromosomal Instability

In the adenoma–carcinoma sequence, histologic progression is accompanied by well‐characterized genetic and epigenetic alterations that are highly correlated with the CIN pathway [10]. CIN is characterized by abnormal changes in chromosome number and structure as well as heterozygous loss of heterozygosity, which can be manifested by partial or whole chromosome deletions, gains, and deletions or insertions of bases. Conventional adenomas progress to carcinoma mainly through successive accumulations of CIN, a pathway that mostly begins with mutations in the adenomatous polyposis coli (APC) gene, which in turn affects Kirsten rat sarcoma viral oncogene homolog (KRAS) activation and mutations in the phosphoinositide 3‐kinase catalytic subunit alpha and SMAD family member 4 genes and subsequent heterozygous deletions of chromosome 18 and loss of TP53 function [11]. APC is thought to be a highly mutated tumor suppressor gene in CRC, with mutations seen in more than 80% of sporadic colorectal tumors [12]. Hyperactivation of the wingless‐related integration site (Wnt)/β‐catenin signaling pathway, which is commonly seen following APC mutations, regulates cell growth and differentiation, making it closely related to CRC development [13]. Activating mutations in KRAS usually occur after APC mutations, and its oncogenic mechanism may be highly related to the sustained activation of rapidly accelerated fibrosarcoma (Raf)–mitogen‐activated protein–extracellular signal‐regulated kinase, phosphoinositide 3‐kinase (PI3K)/protein kinase B (AKT)/mammalian target of rapamycin (mTOR) [14]. The mechanism of carcinogenesis may be highly related to the persistent activation of Raf–mitogen‐activated protein–extracellular signal‐regulated kinase, PI3K/AKT/mTOR. The inactivation of the tumor suppressor TP53 also plays a key role in the progression of adenomas to cancer [15]. TP53 is the most commonly mutated gene in human tumors and induces cell cycle arrest, senescence and apoptosis in response to cellular stress. In addition TP53 is a marker of invasiveness and metastasis in CRC, and TP53 mutations are more common in advanced tumors and are associated with poor prognosis [16].

### 2.2 Microsatellite Instability

MSI is a molecular hallmark of Lynch syndrome, the most common hereditary CRC syndrome, and is also seen in 15% of sporadic CRCs. The MSI pathway is triggered by mutations in the DNA mismatch repair (MMR) genes that result in instability within the microsatellite region. The microsatellite regions are prone to DNA replication errors, and the MMR system is responsible for monitoring and correcting these errors [17]. The MMR system is responsible for monitoring and correcting these errors. When MMR mutations occur, code‐shifting mutations often occur within the repetitive coding sequences of oncogenes and oncogenes, which are closely associated with the development of Lynch syndrome. A large number of code‐shifting mutations occur. Compared with the CIN pathway, the adenoma–carcinoma pathway, in which MSI is involved, APC and TP53 are mutated less frequently in MSI, whereas mutations in tumor suppressor genes, such as transforming growth factor beta receptor 2, are observed in more than 90% of MSI colorectal tumors [18]. Furthermore, sporadic CRC cases are usually accompanied by active BRAF mutations. One of the more typical mutation types is the v‐raf murine sarcoma viral oncogene homolog B1 (BRAF) 600 amino acid substitution mutation from V to E (BRAFV600E), whose high‐frequency mutational properties are thought to be a factor associated with poor prognosis and overall survival in CRC [19]. At the same time, MSI has a disruptive effect on cell cycle arrest or apoptosis, DNA repair, and some other genes encoding proteins that regulate proliferation, thus promoting CRC development [20].

### 2.3 CpG Island Methylator Phenotype

The serrated pathway refers to the progression of serrated polyps (including sessile serrated adenomas and conventional serrated adenocarcinomas, among others) to CRC, which occurs in nearly 20–30% of CRC cases. As another form of precancerous lesion, unlike conventional adenomatous lesions, the serrated pathway is often accompanied by extensive DNA methylation [21]. DNA methylation, an important epigenetic modality, is a chemical modification that occurs when methyl groups are added to cytosine bases. CpG islands, primarily found in gene promoter and exon regions, are rich in CpG dinucleotide clusters composed of cytosine and guanine nucleotides. Aberrant methylation of these CpG islands often leads to the silencing of oncogenes, contributing to cancer development by inactivating tumor suppressor genes and inducing MMR defects through the inactivation of mutl homolog 1 [22]. The prevalence of a highly methylated phenotype in serrated adenomas, compared with hyperplastic polyps, underscores its critical role in the malignant transformation associated with the serrated pathway [23].

BRAF mutations and DNA methylation are closely linked to the pathogenesis of this pathway. In terms of genetic instability, tumors categorized as CIMP^high^ are typically dependent on BRAF mutations, while CIMP^low^ tumors correlate with mutations in the KRAS gene [24]. Notably, BRAF mutations represent an early event in the serrated pathway and have been identified in serrated proliferative anomalous crypt foci [25]. BRAF can activate extracellular signal‐regulated kinase through the mitogen‐activated protein kinase (MAPK) signaling pathway, facilitating its translocation to the nucleus, where it activates transcription factors that regulate gene expression related to cell growth and proliferation.

### 2.4 Other Routes

The progression from normal colorectal epithelial cells to a malignant phenotype is initiated by specific gene mutations, with chronic inflammation serving as a potential catalyst for development under certain conditions. The elevated risk of CRC in patients with Crohn disease and long‐term ulcerative colitis underscores the significant role of inflammatory pathways in both tumor induction and promotion. Inflammatory pathways refer to oncogenic processes activated by chronic inflammation. Research consistently shows that individuals with inflammatory bowel disease, including ulcerative colitis and Crohn disease, face a substantially higher risk of CRC compared with those with other related conditions [23, 26], highlighting the strong link between chronic inflammation and tumorigenesis. Unlike lesions due to the other two pathways (adenomas, sessile serrated polyps), in the inflammatory pathway, massive chronic inflammation often occurs in multifocal flat mucosa that undergoes aberrant hyperplasia and transformation to tumor. The major stages of this pathway include normal cell–atypical hyperplasia–low‐grade heterogeneous hyperplasia–high‐grade heterogeneous hyperplasia–tumorigenesis. Like other types of CRC, related important oncogenic pathways are also important drivers in inflammation‐associated CRC. However, the timing and frequency of specific molecular events differ. In the adenoma–carcinoma sequence, mutations in the APC gene are pivotal for tumor initiation and are frequently observed, while P53 mutations, often influenced by APC and KRAS mutations, mainly contribute to the progression from adenoma to carcinoma. Conversely, in the inflammatory pathway, P53 mutations can occur early during atypical hyperplasia and are found at a high frequency, whereas APC mutations are significantly less common and typically arise only at the tumorigenesis stage [27, 28]. This indicates that APC mutations are not strictly necessary for the initiation of colitis‐associated cancer. Supporting this notion, studies have demonstrated that long‐term chronic inflammation can induce DNA damage and intestinal tumorigenesis even in the absence of external mutagens [29]. This effect may be linked to the excessive production of reactive oxygen species and reactive nitrogen intermediates during chronic inflammatory states. Inflammatory signals activate pathways such as AKT, signal transducer and activator of transcription 3, and nuclear factor kappa‐light‐chain‐enhancer of activated B cells, which are associated with cell survival and proliferation, while also enhancing β‐catenin activity, thus promoting tumorigenesis [30].

Mechanistic studies on the oncogenic processes of CRC have focused on CIN, MSI, and CIMP. The pathological changes and clinical manifestations during cancer progression vary based on the genomic events involved. Importantly, since these three phenomena are not entirely distinct, their overlapping molecular features provide a more holistic understanding of CRC pathogenesis. Traditionally, cancer research has emphasized the accumulation of mutations in oncogenes and tumor suppressor genes as the primary genetic drivers of cancer progression. However, current genomic sequencing data suggest that the key genes implicated in CRC progression are relatively few. This highlights the need for further investigation into how these critical mutations regulate the biological functions of CRC cells [31].

## 3 Epigenetic Modification

The investigation of gene expression signatures linked to genetic instability in CRC progression has been a pivotal focus of CRC research over the past two decades. Notably, the hypermethylation signature found in the promoter regions of tumor suppressor genes indicates that the epigenome may play a crucial role in mediating nongenetic sequence modifications that contribute to the malignant phenotype of CRC. Epigenetic modifications influence chromatin structure and gene expression without altering the DNA sequence itself, with DNA methylation being a prominent early event in CRC progression. In addition, a growing number of experiments have demonstrated the close relationship between other epigenetic regulators, such as histone methylation and acetylation modifications and ncRNA modifications, and the biological behavior of CRC.

### 3.1 DNA Methylation

DNA methylation, often occurring at CpG dinucleotide sequences (i.e., the specific region where guanine and cytosine are linked by a phosphodiester bond), is the most well‐studied epigenetic modification in CRC carcinogenesis. The modification process relies on the DNA methyltransferase (DNMT) to install the methyl group on the carbon atom at position 5 of cytosine to form 5‐methylcytosine. In the normal mammalian genome sequence, DNA methylation is essential for the normal life activities of the organism and plays a role in maintaining genome stability. Since Greger et al. [32] first identified the hypermethylation state of tumor suppressor genes in retinoblastoma tumors in 1989, there has been a growing understanding of the state and level of DNA methylation in the genome‐wide hypomethylation state and promoter region hypermethylation state are recognized as the most typical epigenetic patterns of CRC, driving cancer progression by mediating genetic instability and silent expression of oncogenes. Among them, the hypomethylation state that occurs in the reverse transcriptional transposon long interspersed nuclear element‐1 is the most typical, and its aberrant expression has been recognized as a biomarker of malignant cancer progression [33]. The hypomethylation of the long interspersed nuclear element‐1 retrotransposon serves as a key indicator of malignant progression, showing a negative correlation with genomic stability and a direct link to poor prognosis in CRC patients.

Additionally, aberrant DNA methylation occurs in CpG‐rich regions, or CpG islands, within promoter regions. When the function of the ‘barrier element’ responsible for protecting the promoter region from methylation is impaired, the CpG islands located in the promoter regions of tumor suppressor genes and DNA repair genes often show hypermethylation [34], which has an effect similar to ‘gene mutation’ and prevents transcription factors from binding to the promoter region, thus inhibiting the transcription and synthesis of the relevant genes [35]. Repression of transcription and synthesis of genes. Closure of chromatin structure and functional repression of transcription factors in the promoter region by repressors are also relevant downstream events leading to epigenetic silencing [36, 37]. The CIMP represents a distinct CRC subtype that highlights the impact of DNA methylation on malignant progression, featuring unique molecular mechanisms that confer specific clinical characteristics [38, 39]. In sporadic MSI CRC, high methylation levels of the MMR gene, particularly mutl homolog 1, lead to functional impairment and accumulation of errors, driving CRC onset and progression [40].

### 3.2 Histone Modification

In eukaryotic nuclei, nucleosomes—composed of an octamer of core histones H2A, H2B, H3, and H4—are tightly wrapped around a 147‐base pair DNA fragment [41]. The prominent N‐terminal tails of nucleosomes undergo extensive posttranslational modifications, including acetylation, methylation, and ubiquitination, which play critical roles in regulating various cellular processes. This array of histone modifications forms a “histone code,” facilitating intricate control over genetic information [42]. The interactions and cascading benefits of these modifications enable sophisticated control of genetic information.

#### 3.2.1 Histone Methylation

Histone methylation, first reported in the 1960s, has been extensively studied for its biological significance [43]. As a reversible covalent modification, it involves adding methyl groups to lysine or arginine residues in histone tails, coordinated by histone methyltransferases and demethylases. Histone methyltransferases, divided into lysine methyltransferases and arginine methyltransferases, install methyl groups from S‐adenosyl methionine (SAM) at specific amino acid sites. Lysine methyltransferases can be categorized based on the presence of SET domains, while arginine methyltransferase 1 mediate various forms of arginine methylation [44]. In contrast, demethylases like lysine‐specific demethylases (LSDs) and jumonji C remove these modifications, maintaining the balance of histone methylation, which is crucial for genomic integrity and transcriptional regulation [45]. Methylation at specific histone sites influences transcriptional activity; for example, methylation at H3K4, H3K36, and H3K79 typically correlates with gene activation, while H3K9, H3K27, and H4K20 methylation is often linked to gene silencing [46].

Alterations in histone methylation patterns can significantly affect chromatin dynamics and are implicated in the progression of various cancers, including CRC [47]. In CRC, dysregulation of the PR domain containing 2 transcript and overexpression of retinoblastoma interacting zinc finger protein 2 correlate with malignancy, where retinoblastoma interacting zinc finger protein 2 modulates tumor behavior through the epidermal growth factor (EGF)/epidermal growth factor receptor (EGFR) pathway, enhancing EGF secretion and influencing tumor immunosuppression and angiogenesis [48]. Additionally, the histone methyltransferase SET and MYND domain containing 2 (SMYD2), which targets H3K4 and H3K36, activates the Wnt/β‐catenin signaling pathway, facilitating epithelial–mesenchymal transition and linking histone modifications to DNA methylation processes [49]. Furthermore, SMYD2 is crucial for angiogenesis in CRC, regulated through its downstream target EGF‐like 7 in the notch homolog signaling pathway. Notably, the combination of BAY‐598, a SMYD2 inhibitor, with apatinib shows promise in overcoming apatinib resistance and enhancing antitumor efficacy [50]. AT‐rich interaction domain 3B (ARID3B) is the most widely expressed member of the AT‐rich interaction domain (ARID) family and is involved in gene expression and chromosomal conformation changes as a DNA‐binding protein [51]. Upregulation of ARID3B expression has been found in a variety of human diseases including cancer [52, 53, 54]. ARID3B is mainly involved in determining CRC development and progression around immune escape and stem cell properties, and overexpression of ARID3B facilitates the recruitment of lysine demethylase 4C to the regulatory regions of NOTCH target genes and intestinal stem cell genes, thereby promoting the transcriptional activation of stem cell‐associated target genes through the demethylation of H3K9me3 [55]. Immunohistochemical analyses have demonstrated that the levels of key modifying enzymes and histone methylation marks, such as LSD1 and enhancer of zeste homolog 2 (EZH2), are elevated in normal tissues compared with non‐CRC tissues. This suggests the potential prognostic utility of LSD1 as a biomarker in CRC [56].

#### 3.2.2 Histone Acetylation

In 1964, Vincent Allfrey first identified acetylation modifications on the lysine residues of histones, proposing their regulatory role in transcription [57]. Subsequent research has revealed that histone acetyltransferases (HATs), histone deacetylases (HDACs), and related bromodomain proteins are crucial transcriptional regulators, influencing various cellular processes including chromatin remodeling, the cell cycle, and angiogenesis [58]. HATs facilitate the recruitment of acetyl coenzyme A (acetyl‐CoA) to transfer acetyl groups to lysine side chains, neutralizing the positive charges on histone tails. This process weakens their electrostatic interaction with DNA, enhancing chromatin accessibility and initiating transcription, thus regulating gene expression [59]. Conversely, HDACs promote tighter DNA binding to histones, inhibiting gene transcription. Additionally, regulatory proteins such as HATs, methyltransferases, and ATP‐dependent chromatin remodelers interact with specific chromatin sites through bromodomains to modulate gene transcription [60]. Acetylation can also interact with DNA methylation and other posttranslational modifications, collectively influencing cancer development and metastasis [61, 62]. The dynamic nature of histone modifications during carcinogenesis highlights their potential as drug development targets in CRC [63].

In CRC, abnormal histone acetylation correlates closely with tumor development. For instance, Karczmarski et al. [64] noted significantly increased acetylation of histone H3K27 in CRC tissues compared with normal counterparts. Ashktorab et al. [65] found elevated acetylation of H4K12 and H3K18 in moderately to highly differentiated CRC, while lower levels were observed in poorly differentiated tumors. HDAC2 expression correlated with the progression from adenomas to CRC. Fraga et al. [66] reported a loss of lys16 acetylation in HCT116 cells. Additionally, histone acetylation serves as a critical biomarker for CRC metastasis. One study revealed that the oncogene anterior gradient 2 enhances CRC metastasis by regulating p300/CBP‐associated factor and sirtuin (SIRT)2‐mediated H3K9 acetylation [67]. In patients with heterochronic liver metastases from CRC, H3K9 acetylation levels significantly correlated with tumor histology [68]. A quantitative acetylation proteomics study further characterized the acetylation profiles in primary and metastatic CRC tumors, identifying significant alterations at the H3.2 Lys19 and H2B Lys121 loci [69]. Moreover, histone acetylation modifications hold prognostic value; expressions of SIRT1, HDAC1, and HDAC2, along with levels of H3K56Ac and H4K16Ac, serve as potential prognostic markers for CRC. Survival analyses indicate that patients with higher expression levels of these markers tend to experience better survival rates and fewer tumor recurrences [70].

Recent studies have extensively explored the role of nonhistone acetylation in tumors. This acetylation modification can enhance the sequence‐specific DNA binding of certain nonhistone proteins, including p53, nuclear factor kappa‐light‐chain‐enhancer of activated B cells, and E2F transcription factor. Conversely, it may also reduce DNA binding by other factors such as forkhead box O1, high mobility group protein I, and p65 [71]. These contrasting effects arise from differences in the specific acetylation sites involved. For instance, HDAC3 has been identified as a protein that is upregulated in colon cancer; its overexpression, along with other class I HDACs, suppresses the transcription of the cell cycle mediator p21 in a Sp1/Sp3‐dependent manner [72]. Additionally, nuclear receptor dimerization partner 1, a histone‐binding protein, has been found to be highly expressed in human CRC. This upregulation may regulate HDAC1 expression, influencing both the acetylation and transcriptional activity of p53, thereby impacting the progression of colon cancer [73]. Nonhistone acetylation also plays a critical role in the distal metastasis of CRC through various pathways. Notably, isocitrate dehydrogenase 1 (IDH1) is found to be highly acetylated in both primary tumors and liver metastases of CRC. SIRT2, the primary deacetylase for IDH1, can regulate its deacetylation, subsequently affecting cellular metabolism and inhibiting liver metastasis [74]. Moreover, EGFR has been identified as a key player in liver metastasis. Acetylation of EGFR at the K1037 site, which can be inhibited by thioredoxin, leads to increased phosphorylation of EGFR, thereby activating the MAPK signaling pathway and promoting CRC metastasis [75].

### 3.3 Noncoding RNA

ncRNAs are small RNA molecules that do not code for proteins but play critical roles in human development and various diseases, including cancer. In the past decade, research has significantly advanced our understanding of how ncRNAs contribute to the epigenetic landscape of CRC.

MicroRNAs (miRNAs) are known to regulate posttranscriptional processes in over 60% of human protein‐coding genes [76]. These highly conserved molecules are processed in the nucleus and cytoplasm and bind to the UTR regions of target mRNAs through base complementary pairing. Dysregulation in miRNA biosynthesis often results from mutations or epigenetic modifications that lead to silenced gene expression, either through mRNA degradation or translational repression [77, 78]. Evidence suggests that the expression of certain miRNAs involved in oncogenic pathways correlates with CpG island hypermethylation. For example, miR‐21‐5, a factor in CRC progression, negatively regulates the expression of ten‐eleven translocation 1, with reduced ten‐eleven translocation 1 levels closely linked to tumor growth and metastasis [79]. High methylation of CpG islands is known to contribute significantly to the functional inactivation of miR‐34b and miR‐34c in CRC. Remarkably, demethylation treatments can restore the activity of these miRNAs, coinciding with a marked increase in the levels of H3K4me3 [80].

**FIGURE 2:**
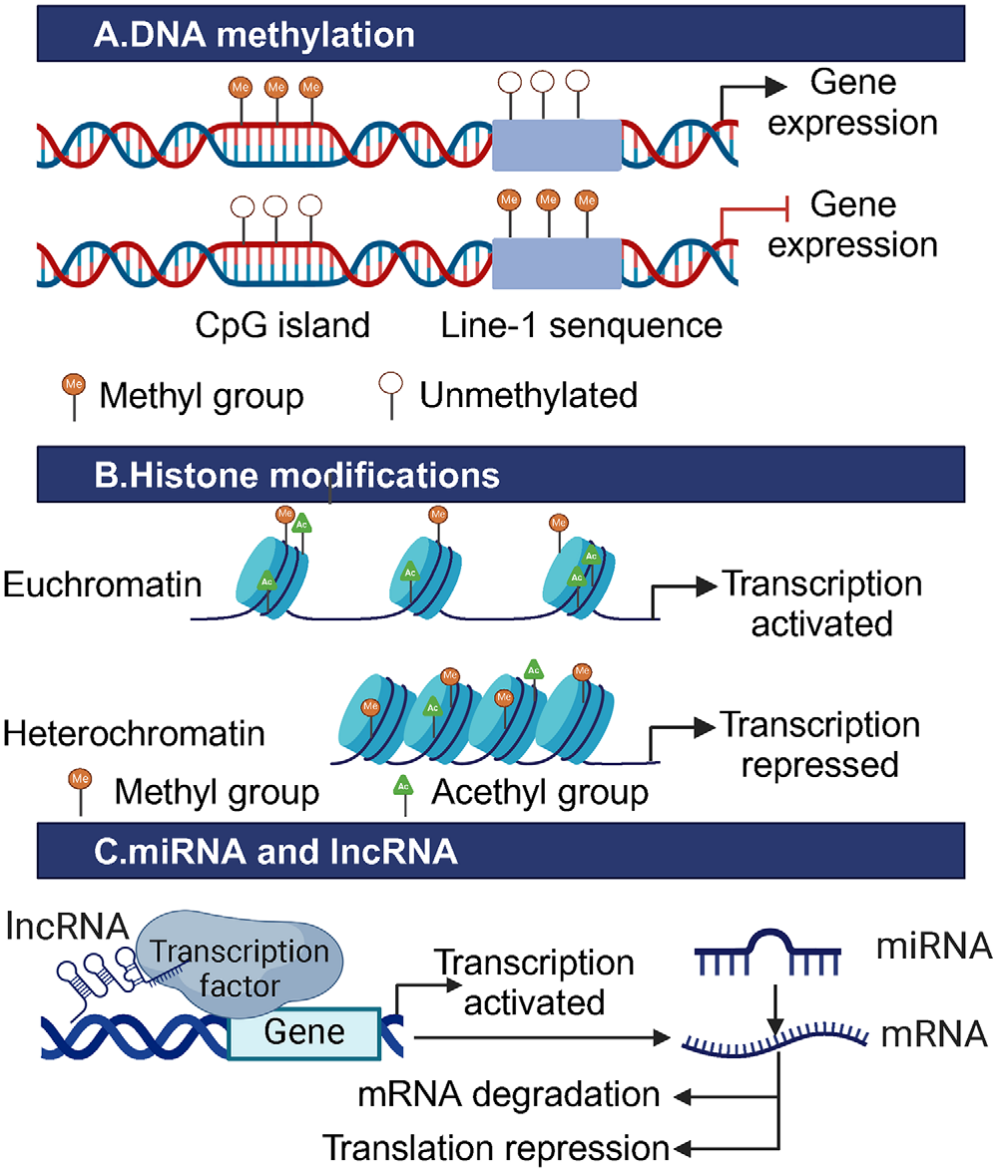
Epigenetic modifications in CRC. Epigenetic modifications influence gene expression by altering chromatin structure. In CRC, a characteristic epigenetic pattern is observed, marked by low methylation across the genome and high methylation in promoter regions. Methylation modifications at H3K4, H3K36, and H3K79, along with high acetylation levels, promote a more relaxed chromatin structure, facilitating transcriptional activation. Conversely, methylation at H3K9, H3K24, and H4K20, combined with low acetylation, leads to transcriptional repression. LncRNAs can recruit transcription factors to modulate gene expression. Additionally, abnormal miRNA‐mediated silencing disrupts signaling pathways and drives the malignant progression of CRC by inducing mRNA degradation and/or inhibiting translation.

Long noncoding RNAs (LncRNAs), which are over 200 nucleotides in length, are increasingly recognized for their role in mediating epigenetic modifications that influence genetic information interpretation. For instance, metastasis‐associated lung adenocarcinoma transcript 1, a lncRNA highly expressed in CRC, interacts with the histone demethylase Jumonji domain‐containing 2C. This binding occurs at the promoter region of metastasis‐associated lung adenocarcinoma transcript 1, leading to an upregulation of metastasis‐associated lung adenocarcinoma transcript 1 levels by reducing site‐specific histone methylation. This mechanism facilitates the proliferation and metastasis of CRC cells [80]. Similarly, elevated levels of HOX transcript antisense RNA contribute to the maintenance of hepatic metastatic capacity in CRC, synergistically supporting malignant transformation through interactions with histone methyltransferase polycomb repressive complex 2, which mediates epigenetic changes [81] (Figure 2).

## 4 Tumor Metabolism

To thrive in a hypoxic and acidic microenvironment, tumor cells undergo significant metabolic reprogramming. This widespread alteration in energy metabolism has emerged as a promising marker for cancer diagnosis [82]. Over the past decade, various analytical techniques have been employed to investigate the metabolic profiles of CRC, revealing unique metabolic characteristics and vulnerabilities compared with adjacent noncancerous tissues. The distinct metabolic adaptations in different CRC subtypes facilitate exponential cell growth and proliferation, offering new insights into the molecular mechanisms underlying CRC [83].

Warburg et al. [84] first noted that tumor tissues consume significantly more glucose than normal tissues, utilizing glycolysis for ATP production even in the presence of oxygen—a phenomenon termed aerobic glycolysis. This observation has sparked extensive research into the aberrant metabolism of tumor cells [84]. Beyond glucose, tumor cells can utilize various substrates to satisfy their growth demands. Core metabolic pathways, including glucose, amino acid, and lipid metabolism, are often reprogrammed in these cells. Moreover, key molecules involved in CRC development, such as Wnt, KRAS, and p53, also function as metabolic regulators, suggesting that alterations in these pathways can significantly influence CRC initiation, progression, and metastasis [9]. The metabolic pathways involved in the development of CRC are also metabolic regulators.

### 4.1 Glucose Metabolism

The Warburg effect is frequently highlighted as a defining characteristic of CRC glucose metabolism. Notably, glycolytic and oxidative phosphorylation processes achieve a metabolic equilibrium in varying environments, often skewed toward glycolysis to rapidly produce energy and intermediates essential for cell growth [85]. Proteomic and metabolomic studies have demonstrated that several key glycolytic enzymes and transporters—such as hexokinase (HK), phosphofructokinase, lactate dehydrogenase (LDH), and glucose transporter (GLUT)—are upregulated in CRC, correlating with poor prognoses [86]. Given the abnormal expression of metabolic regulators during the adenoma–carcinoma progression in CRC, combined with the multifaceted roles of oncogenic signaling pathways in metabolic processes, existing findings suggest that these oncogenic and metabolic signals converge during carcinogenesis, driving the onset and progression of CRC in a complex manner.

**FIGURE 3:**
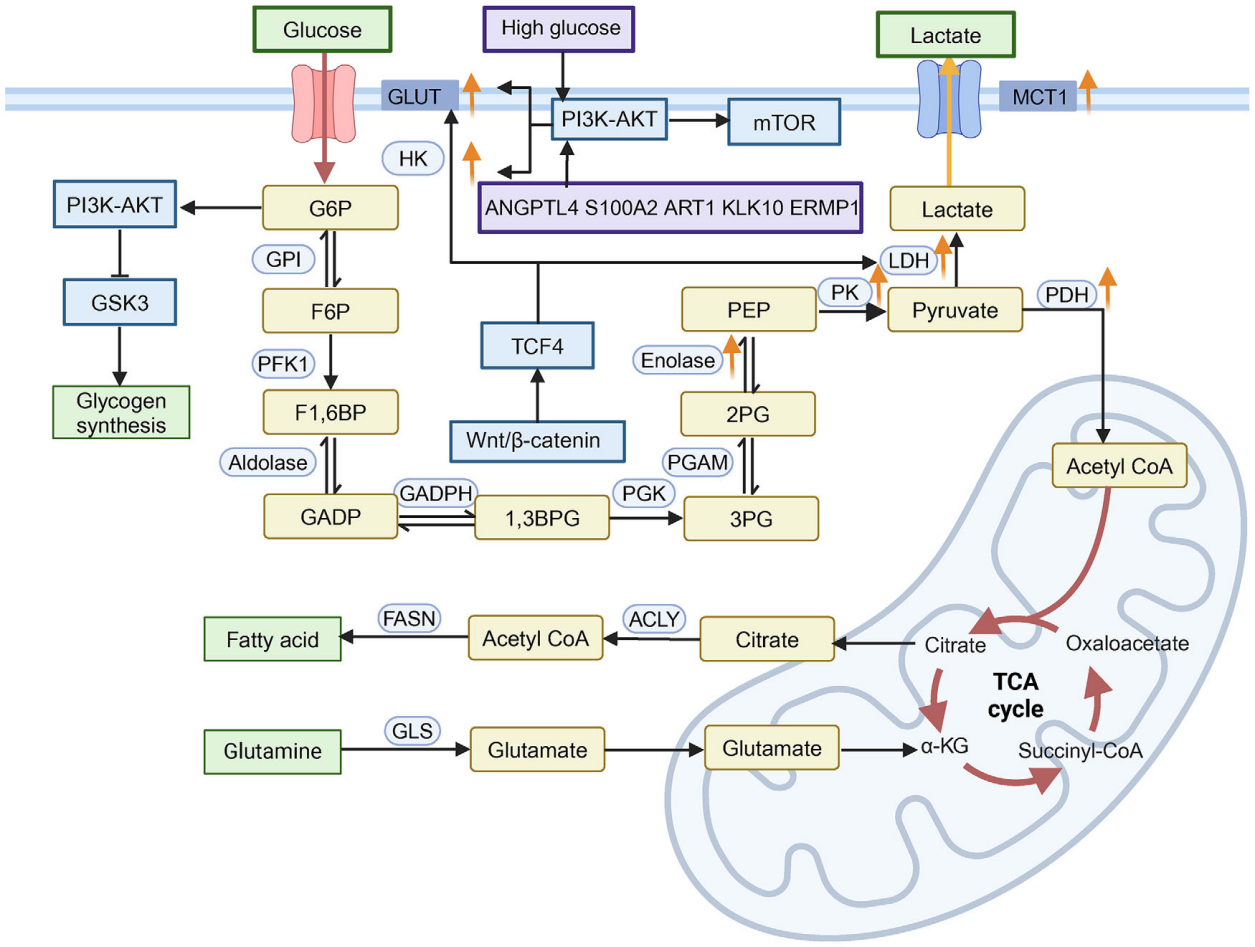
Overview of glucose metabolic reprogramming in CRC. The Warburg effect is a key characteristic of glucose metabolism in CRC, marked by significantly increased glucose uptake and lactate production to satisfy the high energy demands and provide intermediates for rapid cell growth. Gene mutations and downstream signaling pathways play crucial roles in regulating the levels of transporters and key glycolytic enzymes. Accumulated genomic alterations drive the onset and progression of CRC, which is closely associated with poor prognosis. *Abbreviations*: MCT1, monocarboxylate transporter 1; G6P, glucose‐6‐phosphate; GPI, glucose phosphate isomerase; F6P, fructose‐6‐phosphate; PFK1, phosphofructokinase 1; F1,6BP, fructose 1,6‐bisphosphate; GADP, glyceraldehyde 3‐phosphate; GADPH, glyceraldehyde 3‐phosphate dehydrogenase; 1,3BPG, 1,3‐bisphosphoglycerate; PGK, phosphoglycerate kinase; 3PG, 3‐phosphoglycerate; PGAM, phosphoglycerate mutase; 2PG, 2‐phosphoglycerate; PEP, phosphoenolpyruvate; PK, pyruvate kinase; PDH, pyruvate dehydrogenase; ANGPTL4, angiopoietin‐like protein 4; S100A2, S100 calcium binding protein A2; ART1, ADP‐ribosyltransferase 1; ERMP1, endoplasmic reticulum membrane protein 1; TCF4, transcription factor 4; GSK3, glycogen synthase kinase 3; TCA, tricarboxylic acid cycle; α‐KG, alpha‐ketoglutarate; ACLY, ATP‐citrate lyase; FASN, fatty acid synthase; GLS, glutaminase.

High glucose levels drive the proliferation and growth of a variety of tumors, including CRC. Functionally, higher levels of glucose provide an adequate energy supply for tumor growth, allowing for greater aggressive activity. Mechanistically, a high‐glucose environment induces the expression of GLUT1 and HKII genes while activating the PI3K/AKT/mTOR pathway, promoting CRC proliferation [87]. Additionally, angiopoietin‐like 4, S100 calcium binding protein A2, ADP‐ribosyltransferase 1, and rndoplasmic reticulum membrane protein 1 are implicated as cancer drivers in CRC, primarily by activating the PI3K/AKT pathway, which enhances glucose metabolism reliance in tumor cells, sustaining elevated GLUT1 levels and malignant progression [88, 89, 90, 91]. T Studies indicate a positive correlation between Kallikrein‐related peptidase 10 (KLK10) gene expression and poor prognosis in CRC. Knockdown of KLK10 inhibits glucose metabolism and cell proliferation [92], suggesting that targeting KLK10 could downregulate GLUT1 via the PI3K/AKT/mTOR pathway, thereby inhibiting CRC growth [93]. Hypoxia‐inducible factor 1‐alpha (HIF‐1α), as a regulator of cellular adaptation to hypoxia, has been shown to reprogram CRC glucose metabolism. Under HIF‐1α activation, glycolytic metabolism is enhanced through pyruvate dehydrogenase kinase 1 (PDK1) upregulation, boosting tumor cell proliferation [94]. Recent findings suggest HIF‐1α shifts CRC metabolic processes toward glycolysis and the pentose phosphate pathway (PPP), impacting glucose and lactate utilization in the context of PI3K/AKT and β‐catenin signaling, and increasing CRC sensitivity to 5‐fluorouracil (5‐FU) [95]. The Wnt/β‐catenin signaling pathway is crucial for tumor growth and progression, maintaining the Warburg effect and regulating key metabolic enzyme activity. Mutations in APC trigger a positive feedback loop involving Wnt/β‐catenin signaling and pyruvate kinase M2 (PKM2), enhancing lactate dehydrogenase A (LDHA) and GLUT1 expression [96]. Accumulation of APC mutations, alongside KRAS activation and TP53 inactivation, marks significant genomic changes from normal epithelial cells to CRC. APC mutations occur early, inhibiting glycogen storage by upregulating glycogen synthase kinase 3 function [97]. Furthermore, APC gene alterations can downregulate mitochondrial pyruvate carrier 1 in colorectal adenocarcinoma, disrupting mitochondrial oxidative phosphorylation [98]. KRAS and TP53 also play vital roles in CRC glucose metabolism and energy regulation [99] (Figure 3).

Increased glycolysis is an early metabolic hallmark of CRC, with active glycolytic pathways providing energy and biosynthetic precursors essential for proliferation and metastasis. Understanding these metabolic pathways offers new insights into CRC carcinogenesis.

### 4.2 Lipid Metabolism

**FIGURE 4:**
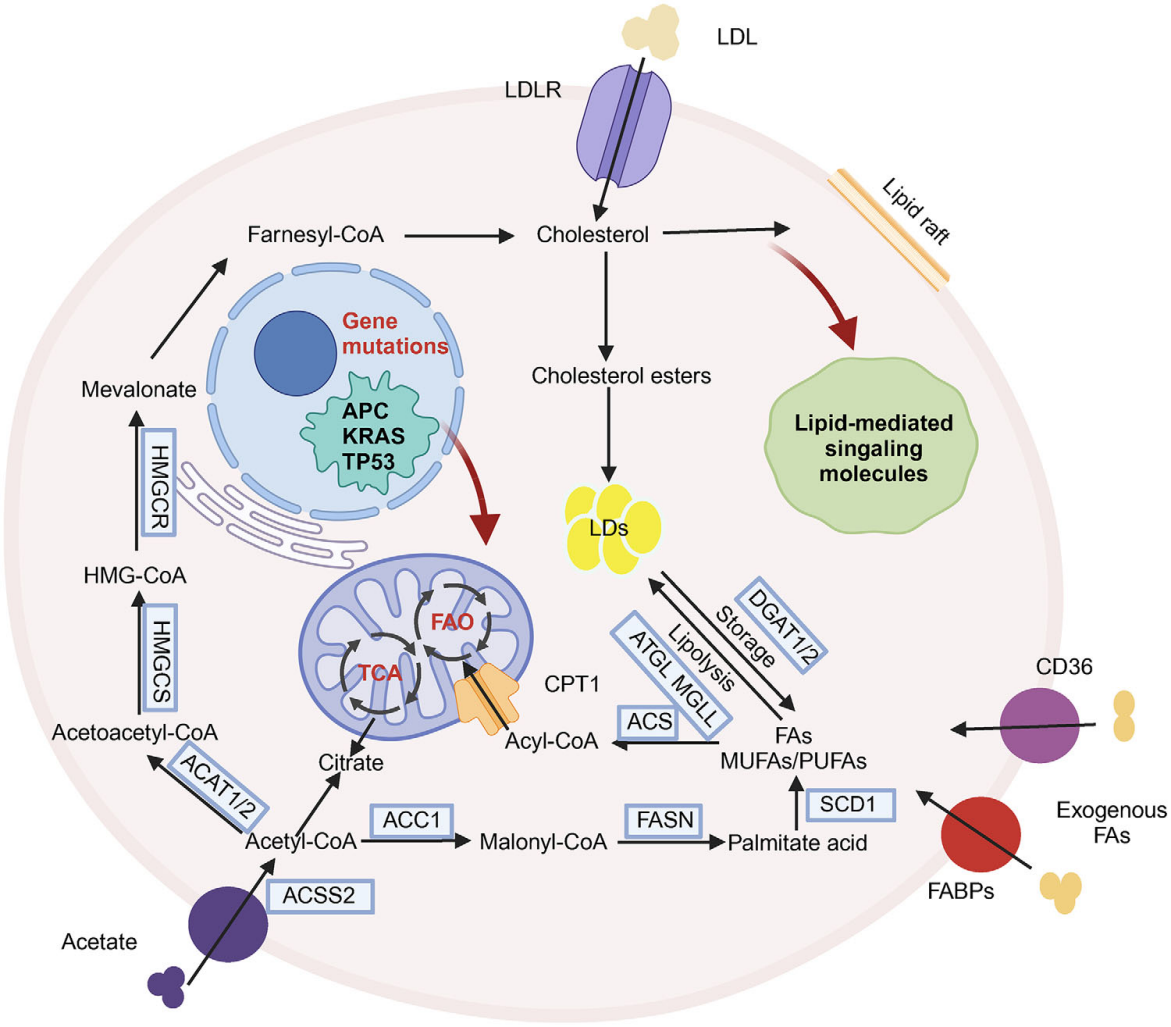
Overview of lipid metabolism reprogramming in CRC. To adapt to the dynamically changing tumor microenvironment, CRC cells undergo genetic mutations that reprogram lipid metabolism. This includes the uptake and production of fatty acids, fatty acid oxidation, cholesterol synthesis, and the formation and breakdown of lipid droplets. Additionally, lipids serve as signaling molecules and essential components for cell membrane production, thereby promoting the survival and progression of CRC cells. The key enzymes involved in lipid metabolism are highlighted in the blue box. *Abbreviations*: LDLR, low‐density lipoprotein receptor; MUFAs, monounsaturated fatty acids; PUFAs, polyunsaturated fatty acids; HMGCR, 3‐hydroxy‐3‐methylglutaryl‐CoA reductase. HMGCS, 3‐hydroxy‐3‐methylglutaryl‐CoA synthase; ACAT1/2, acyl‐CoA, cholesterol acyltransferase 1/2; DGAT1/2, diacylglycerol O‐acyltransferase 1/2; ATGL, adipose triglyceride lipase; MGLL, monoacylglycerol lipase; ACS, acyl‐CoA synthetase.

Several mechanistic aspects of lipid metabolism regulation in CRC have been identified, and advanced metabolic profiling techniques have clarified the lipid metabolic characteristics typical of this condition. Notably, the interplay between abnormal lipid metabolism and CRC has garnered increasing attention (Figure 4).

First, CRC enhances the uptake of exogenous fatty acids. Research has demonstrated that the fatty acid translocase cluster of differentiation 36 (CD36) is highly expressed in human CRC tissues, and its upregulation also promotes CRC metastasis [100, 101]. Fatty acid binding protein (FABP) is an intracellular protein involved in both intra‐ and extracellular lipid metabolism, with multiple isoforms exhibiting altered expression levels in CRC cell lines. FABP5 is prominently expressed across these lines, and its function does not rely solely on its ability to activate CRC cell proliferation and metastasis via the peroxisome proliferator‐activated receptor (PPAR)β/δ pathway [102]. Additionally, the plasma membrane fatty acid binding protein FABP6 is highly expressed in CRC patients and correlates with poor prognosis [101]. Second, CRC upregulates the synthesis of de novo fatty acids. Despite increased exogenous uptake, cancer cells remain significantly reliant on endogenous fatty acid synthesis. Fatty acid synthase (FASN), a key enzyme in de novo lipid synthesis, is also highly expressed in tissues from primary CRC and liver metastases [9]. As a crucial player in lipid metabolism, FASN is involved in glycolysis and amino acid metabolism. Zaytseva et al. [103] conducted a glycolytic stress assay, revealing that glycolysis levels decreased, while glycolytic reserve levels were notably downregulated in the HCT116 and HT29 CRC cell lines following FASN inhibition. ATP‐citrate lyase (ACLY), which converts citrate into acetyl‐CoA, serves as the rate‐limiting enzyme in the initial step of lipid synthesis. It has been shown that ACLY promotes colon cancer metastasis both in vitro and in vivo [104]. Recent findings indicate that Catenin Beta 1 (CTNNB1) may play a role in this process; both ACLY and CTNNB1 are highly expressed in CRC, and their interaction (ACLY–CTNNB1 complex) significantly enhances the transcriptional activity of CTNNB1, facilitating CRC cell proliferation and metastasis [104]. Acetate can also be converted to acetyl‐CoA via acyl‐CoA synthetase short‐chain family member (ACSS) [105]. When lipogenesis pathways are disrupted, the activities of key enzymes involved in fatty acid production often adjust accordingly. This was validated in a study by Zaidi et al. [106], where silencing of the ACLY gene resulted in increased expression of ACSS2 and FASN across all cell lines, demonstrating an acetate‐dependent rise in lipid synthesis. Stearoyl‐CoA desaturase (SCD) is a membrane protein located on the endoplasmic reticulum that catalyzes the conversion of saturated fatty acids to monounsaturated fatty acids, promoting lipid biosynthesis. Additionally, SCD1 regulates the saturated fatty acids to monounsaturated fatty acids ratio, facilitating tumor cell proliferation and metastasis [107]. Further investigations revealed that SCD1 influences CRC proliferation by engaging with the AKT and MAPK pathways [108]. Sterol regulatory element‐binding proteins (SREBP) are vital transcription factors in lipid metabolism, regulating the expression of genes involved in fatty acid, cholesterol, and triglyceride synthesis. SREBPs fulfill the energy requirements of CRC by synthesizing lipids; knocking down SREBP1 or SREBP2 has been shown to inhibit CRC progression and reduce tumor stem cell expression levels [109]. The roles of ACLY and SREBPs in linking glucose and lipid metabolism have been emphasized. After the knockdown of ACLY and SREBPs, CRC cell proliferation, metastasis, and lipid production were inhibited, while apoptosis of cancer cells was enhanced [110]. Adipose triglyceride lipase, a key enzyme in fatty acid metabolism, is highly expressed in CRC. Evidence suggests that adipose triglyceride lipase can promote CRC cell proliferation and growth by inhibiting the mTOR signaling pathway and activating SIRT1 expression, thus regulating the autophagy process, which impacts clinical staging and prognosis in CRC.

Finally, fatty acid β‐oxidation (FAO) is aberrantly activated in CRC. Acyl‐CoA synthetase long‐chain family member 3 plays a crucial role in activating long‐chain fatty acids, providing substrates for β‐oxidation. Elevated expression of Acyl‐CoA synthetase long‐chain family member 3 enhances fatty acid oxidation, leading to increased production of ATP and nicotinamide adenine dinucleotide phosphate (NADPH), which promotes epithelial‐to‐mesenchymal transition and the invasion of CRC cells [111]. Carnitine palmitoyltransferase 1A (CPT1A) is a key rate‐limiting enzyme in fatty acid oxidation. The CPT1A‐mediated fatty acid oxidation pathway has been shown to sustain reactive oxygen species scavenging and enhance reduced glutathione production by increasing NADPH levels. This mechanism protects CRC cells from loss‐of‐nest apoptosis and facilitates cancer cell metastasis by maintaining redox homeostasis.

In cancer cells, excess lipids are converted into cholesterol esters and triglycerides within the endoplasmic reticulum, leading to the formation of lipid droplets (LDs) [112]. Compared with normal controls, CRC stem cells exhibit the highest LD content, which correlates significantly with levels of CD133 and Wnt, suggesting that LD overexpression enhances the oncogenic potential of tumor stem cells [113]. Cholesterol, another vital lipid, stabilizes cell membranes and regulates transmembrane signaling pathways, with low‐density lipoprotein (LDL) serving as the primary cholesterol carrier [114]. Research by Wang et al. [115] demonstrated that LDL receptor levels are significantly higher in CRC tissues compared with normal tissues, correlating with cancer stage. Further studies indicate that LDL can promote CRC progression through the reactive oxygen species‐activated MAPK pathway [115]. Phospholipase A2 group 16, the newest member of the phospholipase A2 family, is involved in key phospholipid metabolic processes. Elevated expression of phospholipase A2 group 16 in CRC tissues, as opposed to normal tissues, has been linked to enhanced CRC proliferation and progression via inhibition of the Hippo signaling pathway, and its levels are inversely correlated with CRC prognosis. Ginsenoside compound K, a natural compound, targets phospholipase A2 group 16 to exert anti‐CRC effects. Additionally, the expression of protein tyrosine phosphatase receptor type O is downregulated in liver metastases of CRC compared with primary tumors. Research shows that silencing protein tyrosine phosphatase receptor type P activates the AKT/mTOR signaling pathway, stimulating the expression of SREBP1 and acetyl‐CoA carboxylase 1 (ACC1), thereby promoting adipose tissue generation. Furthermore, protein tyrosine phosphatase receptor type P silencing can activate the MARK signaling pathway, inhibiting the expression of PPAR1 and acyl‐CoA oxidase 1, which decreases fatty acid oxidation rates, together contributing to the reprogramming of lipid metabolism in CRC [116].

### 4.3 Amino Acid Metabolism

CRC tissues exhibit higher amino acid content compared with normal mucosal epithelial cells and advanced adenoma tissues [117]. Quantitative studies indicate that amino acids, excluding glutamine, serve as the primary carbon source for rapidly proliferating mammalian cells [118]. These amino acids are essential for protein synthesis and provide raw materials for biosynthesis, antioxidant activities, and posttranslational modifications. During CRC pathogenesis, amino acid metabolism adapts, drawing attention to potential therapies targeting glutaminolysis and amino acid metabolism.

Glutamine addiction is a metabolic characteristic observed in many tumors. Tumor cells actively compete for glutamine, utilizing it as a nitrogen source for nucleotide and protein synthesis. Its metabolite, α‐ketoglutarate (α‐KG), participates in the tricarboxylic acid (TCA) cycle and fatty acid synthesis, promoting tumor growth. Transporters like ASCT2 (SLC1A5) facilitate the transfer of glutamine to the cytoplasm and mitochondria, where enzymes such as GLUD1, glutamate–oxaloacetate transaminase 1, glutamate–oxaloacetate transaminase 2, and glutamate–pyruvate transaminase 2 convert it to α‐KG. Lower α‐KG levels can activate the Wnt signaling pathway, sustaining CRC progression and differentiation [119]. KRAS‐mutant CRC shows reprogrammed glutamine metabolism through transporter expression modulation, enhancing energy availability for cell proliferation. Upregulation of SLC7A5 and SLC25A22 activates Wnt signaling, while downregulation of SLC25A21 boosts glutamine metabolism, promoting malignant progression [120, 121, 122]. Targeting glutamine metabolism may provide promising therapeutic avenues for KRAS‐mutant CRC.

Tryptophan, an essential amino acid, is metabolized differently in CRC cells compared with normal colorectal tissues. Most tryptophan is converted to kynurenine, with upregulation of transporters SLC1A5 and SLC7A5 and the metabolizing enzyme amino formyltransferase. Additionally, quinolinic acid, a metabolite of tryptophan, can suppress tumor immunity and facilitate immune evasion [123].

## 5 Interactions Between Tumor Metabolism and Epigenetic Mechanisms

Cancer development involves significant metabolic changes and intricate epigenetic modifications [124]. Tumor metabolic adaptations influence epigenetic alterations through metabolic enzymes and regulatory genes, collectively shaping the CRC epigenetic landscape. Conversely, epigenetic changes provide genetic support for metabolic remodeling, optimizing CRC cell metabolism in varying environments. This interplay between metabolomics and epigenomics elucidates mechanisms sustaining the malignant progression of CRC and opens new targets for diagnosis and treatment.

### 5.1 The Role of Tumor Metabolism on Epigenetic Modifications

Tumor metabolism is intricately regulated, and fluctuations in metabolites often serve as substrates or cofactors for epigenetic modifications. Key metabolites such as acetyl‐CoA, nicotinamide adenine dinucleotide (NAD+), SAM, and α‐KG play critical roles in regulating chromatin dynamics and CRC biology.

#### 5.1.1 Acetyl‐CoA

Recent research underscores the critical role of acetyl‐CoA in lipid metabolism and protein acetylation, highlighting its position as a common substrate that bridges metabolism and epigenetics within a complex regulatory framework [125]. This discussion centers on the interplay between protein acetylation and lipid metabolism reprogramming in CRC, using acetyl‐CoA as a focal point [126]. Acetyl‐CoA metabolism is compartmentalized, with its functions varying by cellular location [127]. In mitochondria, pyruvate, generated from glycolysis via the pyruvate dehydrogenase complex (PDC), is converted into acetyl‐CoA, which enters the TCA cycle [128]. In the cytoplasm, acetyl‐CoA serves as a carbon source for fatty acid and cholesterol synthesis [129]. Additionally, nuclear acetyl‐CoA predominantly modifies lysine residues, playing a vital role in regulating histone modifications that influence tumor growth, proliferation, and metastasis [130].

The metabolism of acetyl‐CoA intricately governs histone acetylation in tumor cells [131]. Carbon‐13 tracing studies have shown that the primary source of acetylated carbon in histone peptides is acetyl‐CoA derived from octanoic acid, illustrating the significance of fatty acid carbon in this process [132]. Lipid metabolic reprogramming dynamically modulates histone acetylation and deacetylation by adjusting the acetyl‐CoA/CoA ratio.

Moreover, lipid metabolism generates key mediators that regulate upstream genes and pathways. Cancer‐related genes, such as AKT, influence acetyl‐CoA levels in cancer cells by enhancing the phosphorylation of ACLY, thereby sustaining acetyl‐CoA production and promoting histone acetylation [131]. Notably, variations in acetyl‐CoA levels affect the selectivity of p300, which alters protein acetylation patterns and subsequently impacts epigenetic regulation [133]. Evidence indicates that acyl‐CoA thioesterase 12 hydrolyzes acetyl‐CoA into acetate and CoA, with both up‐ and downregulation of acyl‐CoA thioesterase 12 affecting protein acetylation and lipid metabolism [134]. Thus, it is plausible to hypothesize that acetyl‐CoA influences histone acetylation levels, driving CRC progression by modulating oncogene expression.

Additionally, acetyl‐CoA derived from glucose and lipid metabolism dynamically regulates protein acetylation kinetics. Under ample glucose conditions, ACLY converts citrate into acetyl‐CoA [135], while the PDC in mitochondria and the nucleus utilizes pyruvate for acetyl‐CoA generation, supporting both TCA cycle and protein acetylation processes [136]. Conversely, under starvation, cancer cells primarily depend on FAO to maintain energy balance [137]. For instance, CRC cells utilize fatty acids from adipocytes to activate mitochondrial FAO, preserving NADPH and ATP homeostasis while mitigating oxidative stress and lipid accumulation [138]. Under glucose deficiency, ACLY and PDC activities are inhibited, prompting AMPK‐mediated phosphorylation of ACSS2, which relocates to the nucleus. There, ACSS2 utilizes acetate from deacetylated nuclear proteins to replenish acetyl‐CoA, thereby adapting to metabolic fluctuations [139].

Emerging evidence indicates that various metabolites can serve as substrates for histone acetylation, notably acetyl‐CoA derived from lipolysis, which hydrolyzes to produce the ketone body β‐hydroxybutyrate (β‐OHB). Research demonstrates that β‐OHB acts as an HDAC inhibitor. Specifically, β‐hydroxybutyrate can induce novel histone modifications at lysine sites 120, 319, and 370 of the p53 gene, resulting in downregulated acetylation levels that inhibit the oncogenic effects of p53 oncogenic effects [140]. Recent studies suggest that β‐OHB exerts dual functions depending on the Warburg effect: in its absence, low concentrations of β‐OHB support ATP generation and cancer cell proliferation without impacting histone acetylation [141]. Conversely, in the presence of the Warburg effect, β‐OHB serves as an epigenetic regulator, inhibiting HDAC class I and II activities [142].

Similarly, butyrate, a gut microbiota derivative, functions as an HDAC inhibitor affecting epigenetic modifying enzymes, thus altering epigenetic landscapes. In studies on HCT116 and HT‐29 CRC cell lines, butyrate demonstrated a dose‐dependent dual effect on proliferation, influenced by the Warburg effect. When the Warburg effect is active, butyrate accumulates in the nucleus, upregulating H3ac levels and inhibiting cancer cell growth. In contrast, in its absence, butyrate promotes lipid metabolism and cell proliferation, while also serving as a substrate for epigenetic inheritance, leading to increased histone acetylation across various cell lines [143]. Additionally, bile acids, regulated by gut microbiota, play roles in lipid metabolism and inflammatory responses. The farnesoid‐X receptor is activated during bile acid metabolism, and its acetylation is linked to inflammation and metabolic diseases [144]. Current research highlights the association between gut microbial‐derived metabolites and CRC occurrence; however, studies exploring the pathogenic roles of gut microbiota in CRC remain limited, suggesting a need for further investigation, particularly with advancing genomic technologies.

Exogenous factors, such as a high‐fat diet (HFD), also contribute to lipid metabolic remodeling and are significant risk factors for colorectal carcinogenesis. Excess lipid intake leads to free fatty acid accumulation, which triggers pro‐inflammatory cytokine secretion and angiogenic factor release, promoting tumor growth [145]. The increased incidence of CRC in HFD‐fed mice, compared with controls, may relate to altered bile acid metabolism and farnesoid‐X receptor activity [146]. Furthermore, HFD affects intestinal flora ecology and compromises the intestinal epithelial barrier, accelerating CRC cell proliferation [147]. In contrast, caloric restriction upregulates SIRT1 levels, while HFD decreases the NAD+/NADH ratio, reducing SIRT activity [148]. This evidence suggests a potential link among HFD, gut microbiota, SIRTs, and CRC, warranting deeper exploration of the underlying mechanisms.

#### 5.1.2 Nicotinamide Adenine Dinucleotide

NAD+ is a crucial metabolite and cofactor that plays a vital role in various metabolic pathways, supporting redox reactions and essential cellular functions. Recent insights highlight the significant influence of NAD+ on histone acetylation modifications. For instance, the metabolic shift of skeletal muscle stem cells from fatty acid oxidation to glycolysis is marked by decreased intracellular NAD+ levels and SIRT1 activity, along with an increase in H4K16 acetylation. SIRT1 acts as a key mediator connecting lipid metabolism and protein acetylation, where lipid metabolism enzymes modulate histone deacetylation through SIRT1, a NAD+‐dependent enzyme activated during energy deficiency [149]. Moreover, NAD+ availability and localized metabolism significantly affect SIRT1‐mediated deacetylation [150]. Fatty acid desaturases are instrumental in maintaining the NAD+ cycle. During glycolysis, NAD+ is converted to NADH, while δ‐5 and δ‐6 desaturases facilitate the synthesis of highly unsaturated fatty acids, converting NADH back to NAD+ and thus sustaining NAD+ levels [151]. Under energy‐rich conditions, a decrease in the NAD+/NADH ratio is accompanied by an inhibition of sirt activity. This situation may lead to histone hyperacetylation and abnormal gene transcription, emphasizing the complex interplay between metabolic pathways and epigenetic regulation [152].

#### 5.1.3 S‐Adenosyl Methionine

One‐carbon metabolism, encompassing the methionine and folate cycles, is essential for nucleotide biosynthesis and protein translation, integrating amino acids such as serine, glycine, and methionine with nutrients like folate.

In the methionine cycle, methionine adenylyltransferase IIα (MAT2A) generates SAM, a crucial methyl donor for epigenetic modifications. SAM facilitates the transfer of methyl groups to DNA, RNA, and proteins, producing S‐adenosylhomocysteine (SAH) as a byproduct. Subsequently, SAH is hydrolyzed to homocysteine, with regeneration of methionine occurring via N5‐methyltetrahydrofolate from the folate cycle. In methionine‐deficient conditions, human pluripotent stem cells exhibit growth inhibition and apoptosis, alongside reduced methionine cycling and downregulation of H3K4me3 markers [153]. SAM and SAH levels reflect the interaction between methionine cycling and chromatin status, with the SAM:SAH ratio correlating positively with methylation status [154]. An experiment has proposed that the methylation repression function of SAH plays an important role in maintaining a hypomethylated state due to its ability to bind more strongly to binding sites [155].

Notably, MAT expression is elevated in CRC tissues and correlates with tumor progression [156]. CUL3 protein regulates MAT2A levels, and downregulation of CUL3 in response to folate deficiency leads to increased MAT2A levels, promoting CRC cell proliferation [157]. Nicotinamide N‐methyltransferase also plays a role in CRC progression by depleting methyl donors during the conversion of SAM to SAH, thereby reducing tumor cell methylation. This metabolic pathway enhances CRC sensitivity to 5‐FU by inhibiting the apoptosis signal‐regulating kinase 1–p38 MAPK pathway [158]. Elevated homocysteine levels can serve as biomarkers for CRC recurrence and prognosis, as they activate DNMT, linking one‐carbon metabolism and methylation status [159].

#### 5.1.4 α‐Ketoglutarate

α‐KG, an intermediate in the TCA cycle and glutamine catabolism, inhibits the differentiation of mouse embryonic stem cells by promoting DNA and histone demethylation [160]. In the TCA cycle, IDH catalyzes the production of α‐KG, and mutations in IDH are associated with various diseases, including CRC [161]. The accumulation of 2‐hydroxyglutarate from IDH mutations antagonizes the activity of histone demethylases and ten‐eleven translocation 1 enzymes, resulting in a hypomethylated genome that drives tumorigenesis [161]. Combined treatment using the IDH1 mutation inhibitor ML309 and the TETase inducer vitamin C in the IDH1 mutant HCT116 cell line showed better synergistic therapeutic efficacy. Functionally, the combination of the two drugs induced apoptosis in tumor cells thus exerting better antitumor activity; mechanistically, ML309 and vitamin C acted together on the key pathways of DNA methylation presentation, significantly reversing the inhibited genome‐wide hypomethylation status while effectively stimulating the expression of tumor suppressor genes [162].

**FIGURE 5:**
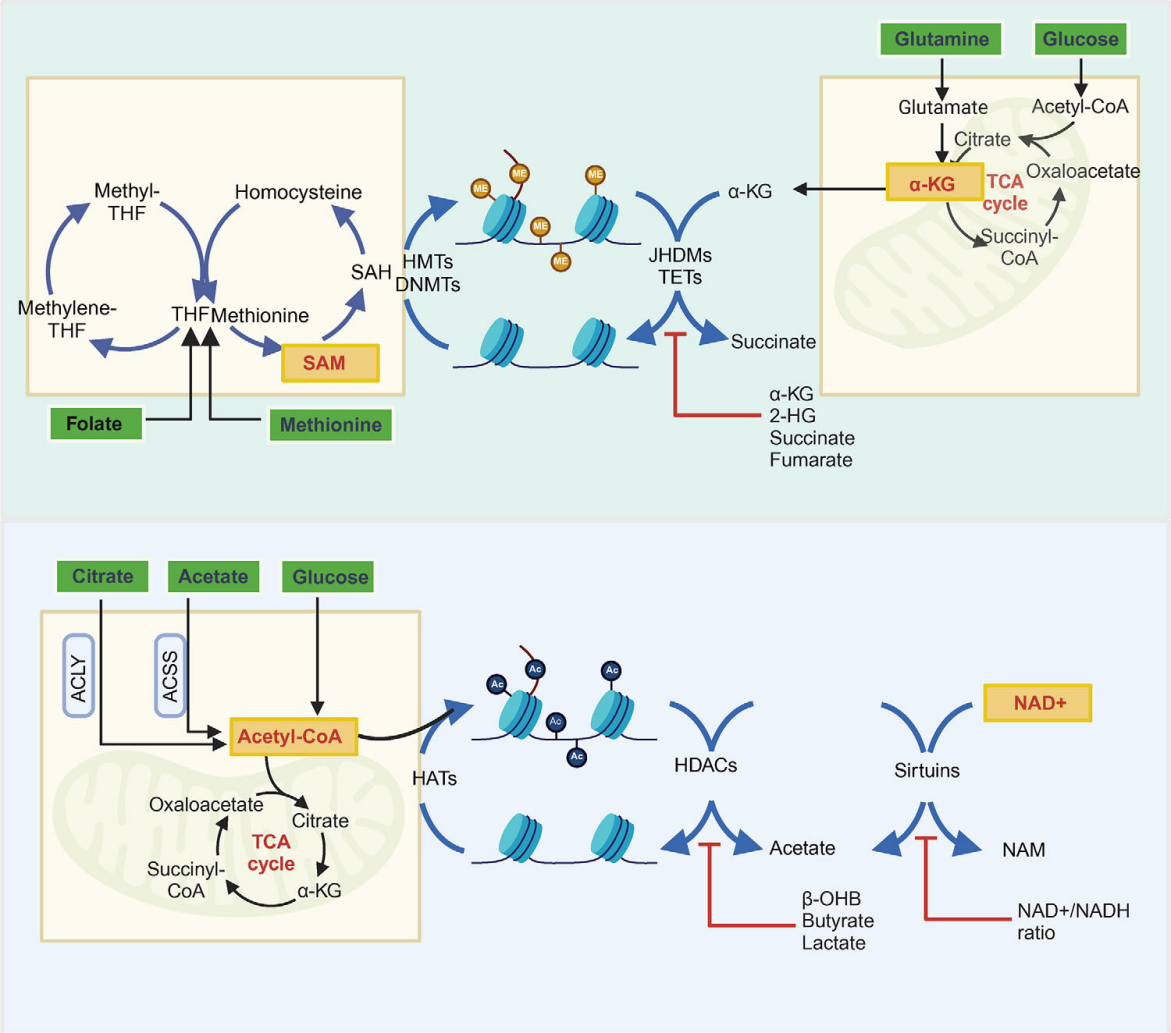
The influence of tumor metabolism on epigenetic modifications. Tumor metabolism, shaped by the tumor microenvironment, involves metabolic enzymes and metabolites that play crucial roles in tumor progression as substrates or cofactors for epigenetic modifications. Key intermediates such as acetyl‐CoA, NAD+, SAM, and α‐KG link epigenetic modifications to metabolic reprogramming. SAM, produced through one‐carbon metabolism, is utilized by HMTs and NDNMTs to add methyl groups to specific sites on DNA and histones, thereby regulating their methylation levels. α‐KG, derived from the TCA cycle and glutamine, serves as a cofactor for JHDMs and TET proteins, facilitating the demethylation of DNA and histones. Acetyl‐CoA, a vital metabolite, regulates histone acetylation and can accumulate through aerobic glucose metabolism, citrate, and acetate metabolism, contributing acetyl groups at specific histone sites. Additionally, intracellular NAD+ levels are positively correlated with SIRT1 activity, which is crucial for maintaining SIRT1‐mediated deacetylation; a decrease in the NAD+/NADH ratio can inhibit SIRT1 activity. *Abbreviations*: THF, tetrahydrofolate; HMTs, histone methyltransferases; JHDMs, jumonji C domain‐containing histone demethylases; TETs, ten‐eleven translocation proteins; 2‐HG, 2‐hydroxyglutarate; NAM, nicotinamide.

The increased demand of tumor cells for nutrients accelerates glutamine uptake, which is vital for α‐KG synthesis. Low glutamine levels in tumors can limit the cofactor availability for histone demethylases, influencing differentiation and stemness in tumor cells [163]. Recent findings suggest that downregulation of SLC25A21 is associated with poor CRC prognosis and drug resistance, as it reduces α‐KG exocytosis and inhibits DNA demethylase activity [122] (Figure 5).

In summary, metabolic remodeling in CRC is influenced by oncogene activation, metabolic enzyme expression, nutrient availability, gut microbiota, and diet. Variations in metabolites play a critical role in shaping the epigenetic landscape of CRC, affecting chromatin conformation and the expression of chromatin‐modifying enzymes.

### 5.2 The Role of Epigenetic Modifications on Tumor Metabolism

**FIGURE 6:**
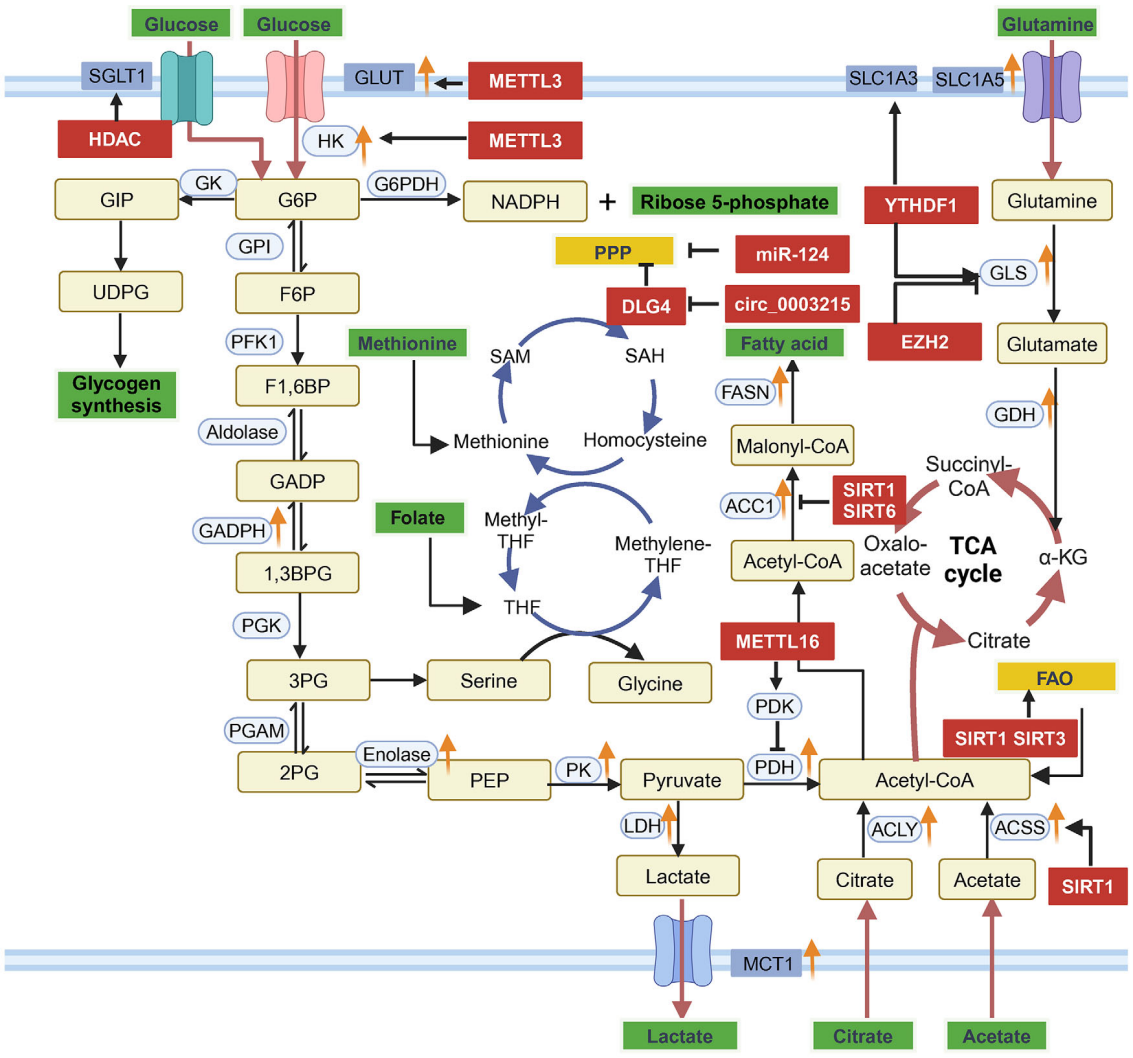
The influence of epigenetic modifications on tumor metabolism. Epigenetic modifications are recognized as key drivers of metabolic reprogramming in tumor cells. Alterations in the levels of crucial enzymes involved in DNA methylation, histone modification, and noncoding RNA have been observed in the disrupted processes of glucose and amino acid metabolism. These changes facilitate the adaptive use of energy and resources by cells in nutrient‐deficient environments, including lipid metabolism. By regulating metabolic phenotypes, epigenetic modifications reveal metabolic vulnerabilities that can serve as potential targets for CRC treatment. *Abbreviations*: SGLT1, sodium‐glucose linked transporter 1; SLC1A3, solute carrier family 1 member 3; GIP, glucose‐dependent insulinotropic polypeptide; UDPG, uridine diphosphate glucose; GK, glucokinase; G6PDH, glucose‐6‐phosphate dehydrogenase; GDH, glutamate dehydrogenase; METTL3, methyltransferase like 3; DLG4, discs large homolog 4;miR‐124, microRNA‐124; circ_0003215, circular RNA 0003215; YTHDF1, YTH domain family 1; EZH2, enhancer of zeste homolog 2.

Recent years have seen an increasing body of evidence that epigenetic modifications significantly regulate tumor metabolism. These epigenomic changes, which accumulate during carcinogenesis, prompt tumor cells to adopt alternative metabolic patterns, creating both metabolic dependencies and vulnerabilities. A deeper understanding of these epigenetic alterations can shed light on the molecular mechanisms underlying metabolic reprogramming in CRC (Figure 6).

#### 5.2.1 Epigenetic Regulation of Glucose Metabolism

Over the past decade, methylation modifications have emerged as crucial mediators of aerobic glycolysis in CRC. In CRC cell lines, the upregulation of the oncogenic factor caveolin‐1 enhances glucose uptake and provides a growth advantage by stimulating GLUT3 expression. Hypomethylation at the promoter CpG site has been identified as a potential trigger for the increased caveolin‐1 expression [164]. Methyltransferase 3 (METTL3), the primary methyltransferase responsible for N6‐methyladenosine (m^6^A) modification, has been linked to aggressive aerobic glycolytic processes in CRC. By adding methylation modifications to HK2 and SLC2A1 (GLUT1), METTL3 supports glycolytic pathway activation [165]. Disruptions in METTL3 levels and the expression of glycolytic enzymes have been shown to influence CRC cell sensitivity to 5‐FU. Mechanistically, METTL3 promotes lactate production from pyruvate; when METTL3 is knocked down, LDHA‐mediated glycolysis is inhibited, reducing chemoresistance to 5‐FU in CRC cells [166]. Another m^6^A methyltransferase, METTL16, further enhances glucose metabolism in CRC by binding to suppressor of G2 allele 1 mRNA, leading to the upregulation of the downstream pyruvate dehydrogenase kinase 4 (PDK4) protein and facilitating glucose uptake [167].

Histone methyltransferases such as EZH2 and EZH1 install methylation marks at H3K27, with their elevated expression correlating with poor prognosis in CRC. Aberrant activation of EGFR is also a key driver of CRC progression. In models where both EGFR and EZH2 are inhibited, the malignant progression of CRC is significantly suppressed, suggesting that the antiproliferative effects of EGFR inhibition may stem from decreased EZH2‐mediated histone methylation [168]. Moreover, HDAC inhibitors have been shown to inhibit EGFR, as well as reduce the expression of the GLUT sodium‐glucose cotransporter 1, thereby impairing CRC cell glucose uptake [169].

The PPP, a critical branch of glycolysis, generates NADPH and ribulose‐5‐phosphate from glucose‐6‐phosphate through glucose‐6‐phosphate dehydrogenase. This pathway provides essential precursors for lipogenesis and nucleic acid biosynthesis. In nutrient‐deficient environments, rapidly proliferating tumor cells adapt by enhancing PPP activity, a process linked to tumor heterogeneity [170]. miR‐124 regulates the PPP by negatively impacting the expression of metabolic enzymes involved in CRC progression [170]. Another study revealed that the expression of m^6^A‐modified circ_0003215 is downregulated in CRC, acting as a connector between epigenetic regulation and metabolic remodeling. Repressed circ_0003215 upregulates miR‐663b and its target discs large homolog 4, which modulates glucose‐6‐phosphate dehydrogenase ubiquitination, thus influencing PPP reprogramming in tumor cells [171]. Given the pivotal role of the epigenome in regulating PPP enzymes, targeting these regulatory genes may present new therapeutic avenues for CRC treatment.

#### 5.2.2 Epigenetic Regulation of Lipid Metabolism

Both genes and histone acetylation are crucial in the reprogramming of lipid metabolism. Mutant oncogenes and tumor suppressors directly affect tumor metabolism, with studies illustrating the connection between oncogenic KRAS genotypes and CRC metabolism [172]. Histone acetylation significantly influences the transcription of metabolic enzymes and key downstream signaling factors, reshaping lipid metabolism in CRC. The upregulation of de novo fatty acid synthesis is a hallmark of cancer metabolism, enhancing membrane integrity and energy supply for critical cellular functions [173]. This synthesis involves the catalysis of acetyl‐CoA by enzymes such as ACSS2 and ACLY, and the carboxylation of acetyl‐CoA to malonyl coenzyme A by ACC, subsequently promoting fatty acid elongation through FASN. Thus, these lipid metabolizing enzymes play a key role in acetyl‐CoA‐mediated lipid metabolism in CRC [174].

In CRC, ACSS2 promotes acetyl‐CoA synthesis by acquiring acetic acid, differing from the short‐chain fatty acid utilization observed in normal colorectal tissues [175]. Notably, ACSS2 is found in the nucleus of certain cancer cells, where it maintains histone acetylation under hypoxic and nutrient‐limited conditions by recapturing acetate released during deacetylation. In various tumor cell lines, including colon tumor 26, ACSS2 acts as a bidirectional enzyme, with its expression enhanced under hypoxic conditions, thus stabilizing acetyl‐CoA and modulating lipid synthesis [176]. Acetyl‐CoA produced in mitochondria cannot cross the inner mitochondrial membrane; instead, it combines with oxaloacetate to form citrate, which is then converted back to acetyl‐CoA in the cytoplasm via ACLY [135]. In patients with liver metastases from CRC, elevated levels of the exosomal gene HSPC111—derived from colorectal cells—alter lipid metabolism in fibroblasts by phosphorylating ACLY, thereby increasing acetyl‐CoA production. This acetyl‐CoA can enter the nucleus, promoting histone acetylation and the expression of C‐X‐C motif chemokine ligand 5, which in turn facilitates organ‐specific translocation of CRCs [177]. In mouse tumor models, including HCT116 and IMR90, metabolic stress can trigger caspase‐10‐mediated cleavage of ACLY, which regulates acetyl‐CoA levels and inhibits general control nonderepressible 5‐mediated acetylation of histones H3 and H4, thereby affecting lipid metabolism. ACC catalyzes the irreversible carboxylation of acetyl‐CoA to generate malonyl coenzyme A, which feeds into the lipogenic pathway through ACC1 and FASN [178]. Enzymes such as ACLY, ACSS2, and ACC1 are widely upregulated in various cancers, including CRC, underscoring their critical roles in the metabolic hub centered on acetyl‐CoA.

Additionally, exploring the effects of lipid metabolism through the lens of acetyl‐CoA under epigenetic regulation has revealed that histone‐modifying enzymes, such as members of the SIRT and HDAC families, mediate the expression levels of lipid metabolic genes. Histone deacetylation can regulate various lipid metabolic pathways, while acetylation impacts multiple aspects of lipid metabolism. Recent studies have indicated that HDAC, particularly HDAC11, is involved in adipogenesis regulation, with HDAC11‐deficient mice displaying hypercholesterolemia [179]. HDAC inhibitors can disrupt the feedback mechanism regulating cholesterol 7‐α‐hydroxylase 1, a key enzyme in bile acid synthesis, leading to increased conversion of LDL cholesterol to bile acids and reduced plasma cholesterol levels [180]. Many members of the SIRT family have been shown to be involved in the regulation of lipid metabolism by modulating histone acetylation. Experimental evidence suggests that SIRT3 and SIRT5 regulate FAO through deacetylation [181]. For instance, SIRT3‐deficient mice exhibit hyperacetylation of long‐chain acyl coenzyme A dehydrogenase under fasting conditions, impairing FAO [182]. Similarly, SIRT1 is crucial for mediating glycolipid metabolism in CRC by deacetylating β‐catenin and promoting FAO under glucose‐deficient conditions [183]. During glucose starvation, significant reductions in acetyl‐CoA correlate with decreased H3K9ac levels, leading to selective activation of lipid metabolism genes through the reduced activity of potassium‐dependent protein 3 and the general control of amino acid synthesis protein 5 [184]. SIRT6 also regulates the deacetylation of various glycolipid metabolism‐related genes, with SIRT6‐deficient mice showing upregulation of triglyceride synthesis genes, contributing to fatty liver disease development [183]. In acidotic tumor cells, both fatty acid synthesis and oxidation occur concurrently, likely due to the downregulation of ACC2 expression mediated by the HDAC, SIRT1 and SIRT6 [185]. This suggests that histone‐modifying enzymes may serve as significant regulators, influencing the metabolite pool and coordinating gene expression in response to nutritional status and intracellular signaling molecules. The relationship between the SIRT family and lipid metabolism in CRC warrants further investigation. Key themes to explore include the interplay among SIRT family members during metabolic processes and their potential synergy with intestinal flora in regulating metabolism. Thus, more research is essential to elucidate the intricate connections between SIRT activity and lipid metabolism in CRC.

While current studies on protein acetylation predominantly focus on histone modifications, numerous proteomic analyses have revealed that nonhistone proteins also undergo acetylation, implicating various transcription factors in metabolic regulation governed by these histone‐modifying enzymes. Notably, SIRT1, the most extensively studied member of the SIRT family, has been linked to metabolic interactions with transcription factors over the past decade. SIRT1 has been shown to inhibit p300‐mediated activation and acetylation of transcription factors such as p65, p53, and forkhead box class O [186]. Additionally, as a regulator of endothelial nitric oxide synthase, SIRT1 exerts antiatherosclerotic effects by mitigating lipid and cholesterol accumulation in endothelial cells [187]. In adipose tissue, it promotes adipogenesis by enhancing the transcription of PPARα and SREBF1c [188]. In the liver, SIRT1 regulates liver X receptor activity, activating ATP‐binding cassette transporter A1 transcription and thereby increasing cholesterol efflux from cells. This perspective prompts further exploration into the broader impacts of histone‐modifying enzymes on other nuclear receptor activities [189]. Importantly, SIRT1 also deacetylates lipid‐metabolizing enzymes, facilitating accelerated lipogenesis through the deacetylation of ACSS1 [190]. Overall, while studies on nonhistone acetylation provide crucial insights into lipid metabolism, research specifically examining the regulation of lipid metabolism via nonhistone acetylation in CRC remains limited, highlighting an urgent need for detailed investigations into this vital connection.

#### 5.2.3 Epigenetic Regulation of Glutamine Metabolism

Under glucose‐deficient conditions, tumor cells depend on glutamine catabolism to supply substrates for the TCA cycle. When glutamine availability is compromised, CRC cells often activate autophagy to manage amino acid levels and maintain intracellular homeostasis [191]. The roles and molecular mechanisms of epigenetic modifications in CRC glutamine metabolism are increasingly being elucidated through ongoing experiments. For instance, the m^6^A modification “reader” YTH domain family 1 (YTHDF1) enhances the translation of the downstream target gene GID complex subunit 8 homolog, promoting the uptake and utilization of glutamine by activating key enzymes such as solute carrier family 1 member 3 and glutaminase (GLS) [192]. Increased activity of solute carrier family 1 member 3 and GLS accelerates glutamine metabolism, driving more aggressive CRC progression [192]. Furthermore, histone methylation mediated by EZH2 under glucose‐deficient conditions has been shown to inhibit GLS expression, exerting tumor‐suppressive effects [193]. The activation of heat shock factor 1 (HSF1) under stress conditions has also been implicated in malignant transformation and poor prognosis in various solid tumors, with recent studies highlighting its role in glutamine metabolism. In vitro studies have demonstrated that silencing HSF1 inhibits CRC cell growth, correlating with reduced glutamine catabolism. Mechanistically, HSF1 recruits DNMT3a, which suppresses miRNA137 targeting of GLS1, thereby promoting CRC tumorigenesis [194]. Given the significant roles of GLS1 and methylation modifications in regulating glutamine energy metabolism, targeting the activities of methylation‐modifying enzymes and their upstream regulators may present a promising strategy for influencing glutamine catabolism in CRC.

In summary, epigenetic modifications play a critical role in regulating cellular metabolic phenotypes and elucidating the mechanistic basis of the modifiable nature of tumor metabolism. There is considerable overlap between metabolism and epigenetics in CRC progression in response to both endogenous and exogenous stimuli. Epigenetic modifications are essential for bridging genetic information and metabolism, contributing to the heterogeneous and adaptive metabolic profiles and therapeutic responses of tumor cells. Fluctuations in metabolite levels from tumor cell metabolism constitute the metabolic pool, influencing the availability of epigenetic regulators and the extent of epigenetic modifications. It is important to investigate how variations in metabolite levels may selectively impact the levels of chromatin‐modifying enzymes, potentially due to differing catalytic properties among these enzymes. This observation underscores the intricate interplay between epigenetics and metabolomics.

## 6 Targeted Therapies

Over the past two decades, numerous studies have endeavored to uncover the molecular mechanisms governing the onset and progression of CRC. Insights gained from genetics and epigenetics have laid a crucial foundation for developing biomarkers and translating these discoveries into clinical applications. Currently, targeted therapies are the primary approach for treating CRC, addressing existing challenges by focusing on key genes and signaling networks implicated in oncogenic and metabolic pathways during the malignant transformation of CRC. This paves the way for more effective predictive, diagnostic, and prognostic markers.

### 6.1 Targeting Cancer‐Causing Genes

Specific oncogenic genetic alterations are significant drivers of CRC progression, making the targeting of these signaling pathways a precise therapeutic strategy for personalized treatment.

The combination of vascular endothelial growth factor inhibitors and EGFR inhibitors serves as a first‐ or second‐line treatment option to induce regression of metastatic CRC [195]. Bevacizumab was the first vascular endothelial growth factor inhibitor approved for this indication. Recent trials have demonstrated that combining bevacizumab with anlotinib, a tyrosine kinase inhibitor, synergistically targets angiogenesis and inhibits the metastatic invasion of CRC cells [196]. A phase III trial showed that the combination of FOLFOXIRI (folinic acid, 5‐FU, oxaliplatin, and irinotecan) with bevacizumab leads to prolonged progression‐free survival and improved prognosis compared with FOLFOXIRI alone [197]. Another promising target for metastatic CRC treatment is ramucirumab. In a randomized phase II clinical study involving advanced KRAS wild‐type CRC, adding ramucirumab to irinotecan and cetuximab resulted in extended progression‐free survival, confirming its therapeutic efficacy in clinical practice [198].

EGFR expression is notably upregulated in CRC tissues and, upon binding to EGF, activates intracellular signaling cascades involved in tumor cell polarization, proliferation, and autophagy [199]. Consequently, targeting EGFR represents a valuable therapeutic strategy. Currently, cetuximab and panitumumab are the two approved EGFR inhibitors for advanced CRC, while additional anti‐EGFR monoclonal antibodies are under investigation [200]. Cetuximab primarily exerts its antitumor effects by inhibiting angiogenesis and metastasis, as well as interfering with the cell cycle. The combination of FOLFOX or FOLFIRI regimens with cetuximab has proven effective in enhancing progression‐free survival and objective response rates in CRC patients [201]. Panitumumab, a fully humanized antibody, demonstrates high affinity for EGFR and has shown greater therapeutic benefit in metastatic CRC compared with the chimeric cetuximab. However, panitumumab combined with various chemotherapeutic agents can lead to increased skin toxicity [202]. Notably, cetuximab and panitumumab have not significantly affected survival rates in CRC with KRAS exon 2 activating mutations, indicating that the efficacy of EGFR inhibitors is primarily observed in KRAS wild‐type CRC [203].

Human epidermal growth factor receptor (HER)2, a member of the HER family, is closely associated with the pathological features and progression of CRC. Targeting HER2 and its downstream regulators is vital to overcoming drug resistance associated with EGFR inhibitors [204]. Trastuzumab and pertuzumab have been approved for use with chemotherapeutic agents in treating HER2‐positive CRC. Trastuzumab deruxtecan has shown efficacy in patients with metastatic CRC previously treated with two or more therapies, resulting in tumor shrinkage and extended survival, albeit with gastrointestinal and hematologic adverse effects [205]. Ongoing phase II studies are investigating combinations of various HER2 inhibitors, including trastuzumab with lapatinib [206], and trastuzumab with pertuzumab [207], which have shown enhanced antitumor activity and tolerability compared with monotherapy.

The BRAFV600E mutation in CRC is frequently linked to activation of the MAPK signaling pathway, contributing to poor prognosis. Therefore, combining BRAF‐targeted therapies with other treatments has emerged as a viable strategy for patients with metastatic CRC. Notably, the combination of encorafenib, binimetinib, and cetuximab has demonstrated superior antitumor efficacy compared with standard treatments [198]. The mitogen‐activated protein inhibitor cobimetinib combined with the BRAF inhibitor SGI‐110 may offer significant tumor growth inhibition in the early stages of CRC treatment [208]. The SEAMARK study validated that combining a BRAF inhibitor (encorafenib), an EGFR inhibitor (cetuximab), and immunotherapy (pembrolizumab) provides a promising therapeutic direction for BRAF‐mutant CRC, ensuring both efficacy and safety in clinical trials [209].

### 6.2 Targeted Epigenetic Modifications

Epigenetic alterations play a significant role in the malignant transformation of benign adenomas, the invasive progression of CRC, and resistance to treatment. Unlike irreversible genetic changes, the reversible nature of epigenetic modifications makes the development and application of epigenetic drugs particularly promising. Two main classes of epigenetic modifiers are DNMT inhibitors and HDAC inhibitors, which can potentially benefit CRC patients by reversing harmful epigenetic changes.

#### 6.2.1 DNA Methylation Inhibitors

Inhibitors targeting DNMTs have demonstrated significant therapeutic potential in both preclinical and clinical studies. For example, decitabine (DAC) inhibits methyltransferase activity and reactivates tumor suppressor genes silenced by promoter methylation. Pharmacological analyses indicate that combining DAC with standard chemotherapeutics like 5‐FU or oxaliplatin results in enhanced efficacy for CRC treatment [210]. Notably, low doses of DAC can stimulate proliferation in the HT‐29 cell line, attributed to the restoration of BCL2 expression [210]. In a phase I/II trial involving refractory CRC, the injectable azacitidine combined with CAPOX (capecitabine and oxaliplatin) showed improved disease stabilization in CIMP^high^ CRC patients [211]. Furthermore, the first epigenetic combination therapy trial, utilizing 5‐azacytidine and entinostat, revealed promising results in extending progression‐free survival for some CRC patients [212]. However, evidence from a phase II trial evaluating guadecitabine (SGI‐110) in combination with irinotecan remains inconclusive, indicating a need for further investigation [5].

#### 6.2.2 Histone Demethylase Inhibitors

Abnormal expression of HDACs is commonly observed in various tumors, making them a significant focus for innovative anticancer therapies. HDACs play a crucial role in regulating the acetylation of numerous proteins, including transcription factors and signaling mediators, which influence tumorigenesis, cancer cell proliferation, and immune responses [213]. By modulating acetylation modifications that affect protein stability and interactions, targeting HDACs to correct aberrant acetylation patterns is considered a promising strategy for cancer treatment [71]. Currently, a range of small‐molecule HDAC inhibitors has demonstrated enhanced anticancer activity, both as standalone therapies and in combination with other agents. The antitumor effects of these inhibitors manifest in several ways: they inhibit tumor growth, regulate transcription, promote apoptosis, and increase the sensitivity of cancer cells to chemotherapeutics [63]. Most marketed HDAC inhibitors are used for various hematological cancers, while research into their application for solid tumors like CRC is still largely in the preclinical phase.

HDAC inhibitors can be categorized based on their chemical structures into four groups: hydroxamic acids, benzamides, cyclic peptides, and short‐chain fatty acids. Vorinostat (SAHA), a pan‐HDAC inhibitor, has been shown to significantly inhibit cell proliferation, induce apoptosis, and cause cell cycle arrest in Caco‐2 cell lines [214]. Additionally, SAHA has been reported to reduce oxaliplatin resistance in human CRC cells [215]. Another study indicated that combining vorinostat or panobinostat with 5‐FU effectively inhibits the transcription and expression of thymidylate synthase genes, overcoming TS‐mediated drug resistance [216]. Scriptaid, a less toxic HDAC inhibitor, disrupts the cell cycle and modifies the pl6 gene, which is hypermethylated in CRC, restoring it to an orthochromatin state through histone modification regulation [217]. Romidepsin, a cyclic peptide selective for HDAC1 and 2, significantly inhibits tumor growth in both CRC subcutaneous and colitis‐associated cancer models. It also increases the percentage of FOXP3^+^ regulatory T cells in the tumor microenvironment while modulating the Th1/Th2 cell ratio, thereby affecting immune responses [218]. Axitinib (Apicidin), a novel cyclic tetrapeptide, inhibits p21WAF1/CIPI by activating cyclin‐dependent kinases, which contributes to its HDAC inhibition and subsequent suppression of CRC cell proliferation [219]. Cidarbenamide, a benzamide‐based HDAC inhibitor, primarily targets class I HDAC isoforms 1, 2, and 3, and class IIb isoform 10. It has been shown to inhibit the PI3K/AKT and MAPK/Ras signaling pathways in vitro, promoting cell apoptosis [220]. In xenograft models, cidarbenamide demonstrated promising antitumor activity alone or in combination with 5‐FU [221]. Entinostat (MS‐27‐275), an active benzamide derivative, induces p21WAF1/CIPI and gelsolin independently of the p53 pathway, resulting in significant antitumor effects [222]. Short‐chain fatty acid‐based HDAC inhibitors, including valproic acid, butyrate, and phenylbutyrate, inhibit HDAC1 and HDAC2. Notably, valproic acid also promotes the degradation of HDAC2 and has been shown to reduce the proliferative advantage of APC‐mutated cells, thereby inhibiting tumor progression [223]. Butyrate inhibits HDAC1 and HDAC2 activity [224], which can also produce precursors of acetyl‐CoA via β‐oxidation, leading to histone hyperacetylation [225].

### 6.3 Targeting Metabolites

Nutrient depletion in the tumor microenvironment prompts cancer cells to explore advantageous metabolic pathways, resulting in reprogrammed metabolism characterized by adaptive preferences and vulnerabilities. Developing inhibitors against metabolic targets, particularly in combination with other therapies for CRC treatment, shows significant promise.

#### 6.3.1 Targeting Glucose Metabolism

Currently developed inhibitors targeting GLUT have demonstrated the ability to impede glucose uptake, leading to therapeutic benefits for tumors characterized by elevated glucose metabolism. Phloretin, a natural GLUT2 inhibitor, inhibits the malignant progression of CRC by reducing glucose uptake and activating the p53 pathway [81]. Phloretin effectively blocks over 80% of glucose uptake but exhibits direct cytotoxicity to cells. Fasentin partially inhibits glucose uptake, influences the expression of genes related to glucose metabolism, and enhances tumor cell sensitivity to death ligands by inducing cell‐cycle arrest, although its mechanism requires further investigation [226]. STF‐31, which acts as a dual inhibitor of nicotinamide phosphoribosyltransferase and GLUT, has been shown to inhibit glucose uptake in tumor cells at high concentrations [227].

Another notable inhibitor of glucose metabolism is 2‐DG, which shares structural similarities with glucose, allowing it to enter cells via GLUT. It competitively inhibits HK, thereby disrupting the glycolytic process and exerting antitumor effects. 2‐DG is safe for oral administration and can be used alone or in combination with other therapies. Notably, the combination of 2‐DG and α‐tocopheryl succinate targets both glycolytic and oxidative phosphorylation pathways, demonstrating significant antitumor efficacy in vitro [228]. To address the short half‐life of 2‐DG, novel analogs have been developed and are undergoing clinical trials. WP1122 is an orally bioavailable analog that exhibits 2‐ to 10‐fold greater antitumor activity compared with 2‐DG [229]. Metformin, an oral first‐line treatment for diabetes, also serves as an HK2 inhibitor, reducing glycolytic activity and slowing CRC tumor growth. Importantly, Metformin treatment has been linked to reduced CRC incidence and inhibited metastasis by downregulating pyruvatePKM2 expression and upregulating IDH1 expression, while maintaining a balance between glycolysis and oxidative phosphorylation [230].

Dichloroacetate (DCA) is a novel metabolic inhibitor that enhances aerobic oxidation primarily by targeting PDK. In vitro studies indicate that the combination of DCA and 5‐FU effectively inhibits apoptosis in CRC cells [231]. DCA also restores the sensitivity of CRC cells to oxaliplatin by downregulating miR‐543 [232]. Oxamate, a compound structurally similar to pyruvate, competitively inhibits LDHA, inducing mitochondrial apoptosis. Combination therapies have proven effective for advanced metastatic CRC. The mTOR inhibitor rapamycin exhibits metabolic effects akin to those of glycolysis inhibitors in CRC, and when used in conjunction with oxamate, it synergistically enhances survival rates and therapeutic efficacy in CRC patients [233].

#### 6.3.2 Targeting Lipid Metabolism

Multiple lines of evidence indicate that abnormal activation of key enzymes involved in lipid metabolism is a critical factor in tumor development, proliferation, and metastasis [234]. Regulating the expression of these key molecules in lipid metabolism could serve as an effective intervention against metabolic reprogramming in tumors, making the identification of potential therapeutic targets essential for treatment and prognosis [235]. Although there are currently no clinical lipid therapies for CRC [236], various small molecule inhibitors have demonstrated therapeutic potential by disrupting lipid metabolism and may be used synergistically with other treatments [237].

FASN, a crucial enzyme in fatty acid synthesis, is highly expressed in CRC and correlates negatively with prognosis. This positions FASN as a significant target for tumor therapy [238]. Several FASN‐specific inhibitors, including cerulenin, C75, orlistat, and TVB‐2640, have been shown to induce apoptosis in cancer cells and exhibit therapeutic efficacy across various malignancies [239]. In vitro studies reveal that cerulenin inhibits DNA replication and S‐phase progression, effectively reducing the proliferation of LoVo cells [240]. Furthermore, cerulenin activates the caspase pathway to induce apoptosis in CRC cell lines and inhibits liver metastases in CT26 and CMT93 models [241]. Research by Chang et al. [242] highlighted that cerulenin downregulates energy metabolism in HT29 and LoVo cells while inhibiting the mTOR signaling pathway, impacting the malignant phenotype of CRC. In the context of unresectable CRC, the efficacy of oxaliplatin has been evaluated in numerous clinical trials [243]. The combination of cerulinin and oxaliplatin can reduce side effects such as oxaliplatin neurotoxicity and dosage, and improve long‐term tolerance to the chemotherapeutic agent [244]. C75 is a FASN inhibitor structurally similar to cerulinin [245]. Cerulinin and C75 act directly or indirectly on CPT1 to promote fatty acid oxidation [246]. Both FASN inhibitors have also been shown to significantly reduce food intake and body weight in treated mice [247]. The second‐generation FASN inhibitor, C93, has shown advantages in treating esophageal cancer without the side effects of anorexia or weight loss [248]. Orlistat, another FASN inhibitor, dose‐dependently activates the caspase‐3 pathway, blocking cells in the G1 phase and inhibiting proliferation and fatty acid synthesis in the HT‐29 cell line [249]. Recent studies have demonstrated that exogenous addition of palmitate restores the activity of HT‐29 cells inhibited by orlistat [250]. Novel TVB inhibitors, TVB‐3664, TVB‐3166, and TVB‐2640, have demonstrated antitumor effects in in vivo and in vitro experiments and are now in clinical trials in CRC patients [251]. It has been proposed that TVB‐3166 can disrupt the structure of lipid rafts in the plasma membrane and inhibit oncogenic routes and oncogene expression, thus exerting anticancer activity [252]. Additionally, various natural compounds, such as luteolin and resveratrol, exhibit anticancer properties and FASN inhibition. In the HT‐29 CRC cell line, luteolin block the cell cycle and downregulate antiapoptotic proteins [253]. Resveratrol inhibits the proliferation of human colorectal Caco‐2 cells [254], and long‐term administration has been shown to induce apoptosis and prevent the growth of aberrant crypt foci in rat colon models [255]. Research by Vanamala et al. [256] demonstrated that resveratrol induces apoptosis and inhibits cell proliferation through modulation of the growth factor 1 receptor /AKT/Wnt signaling pathway and activation of p53.

Beyond FASN inhibitors, ACLY and SCD inhibitors play vital roles in lipid metabolism. ETC‐1002, a potent ACLY inhibitor, activates the AMPK pathway, inhibiting fatty acid and cholesterol synthesis; however, clinical studies have primarily noted its impact on cholesterol synthesis [257]. A study indicated that combining ETC‐1002 with the IGFIR inhibitor linsitinib produces a significant synergistic effect in inhibiting CRC metastasis [258]. SCD1, an enzyme responsible for producing monounsaturated fatty acids, is also negatively correlated with CRC prognosis. The novel oral SCD inhibitor T‐3764518 induces apoptosis in CRC xenograft models [259]. Betulinic acid, a potent SCD1 inhibitor derived from birch, has been shown to block the cell cycle at the G2/M phase and inhibit CRC proliferation [260]. Furthermore, another study demonstrated that betulinic acid impairs clonogenic capacity and induces death in cancer stem cells [261].

FAO is also an important link in cancer progression, and inhibition of the key rate‐limiting enzyme in this link, CPT1, reduces acylcarnitine production [262]. Etomoxir, an irreversible CPT1 inhibitor, significantly inhibited fatty acid uptake and ATP synthesis without affecting tumor cell stemness and angiogenesis [263]. Additionally, a study demonstrated that combining etomoxir with cisplatin enhances the apoptotic effects of cisplatin on HCT116 colorectal cells in a dose‐dependent manner [264]. Another CPT1 inhibitor, perhexiline, was originally developed and utilized for the treatment of angina pectoris [265]. A study by Wang et al. [266] found that oxaliplatin in combination with perhexiline promoted apoptosis in cancer cells and enhanced the sensitivity of HCT116 cells to oxaliplatin.

Statins, commonly used to lower cholesterol, have been linked to CRC development and prognosis [267]. Lovastatin has demonstrated the ability to inhibit both canonical Wnt signaling and alternative pathways (YAP/TAZ), thereby impeding CRC progression [268]. Low‐dose lovastatin induces CRC cell differentiation and significantly increases the sensitivity of CRC cells to 5‐FU [269]. Thus, lovastatin emerges as a potential CRC inhibitor, complementing the established role of statin therapy.

#### 6.3.3 Targeting Glutamine Metabolism

GLS1 catalyzes the conversion of glutamine to glutamate, and its upregulation is closely associated with poor prognosis in various cancers. Targeting GLS1 can disrupt glutamine uptake and utilization, thereby inhibiting tumor progression. However, diazo‐O‐norleucine, the first GLS1 inhibitor, has proven ineffective therapeutically due to significant gastrointestinal side effects [270]. Bis‐2‐(5‐phenylacetamido‐1,2,4‐thiadiazol‐2‐yl)ethyl sulfide, a reversible inhibitor of GLS1, poses challenges for clinical study due to its metabolic instability and low solubility [271]. A novel GLS1 inhibitor, CB‐839, has recently undergone clinical evaluation. While CB‐839 alone has not demonstrated significant therapeutic effects on CRC, its combination with other agents has shown synergistic potential. A phase II trial indicated that coadministering CB‐839 with capecitabine (an oral prodrug of 5‐FU) achieved a progression‐free survival of over 6 months in 21.8% of patients by upregulating neutrophil extracellular traps [272]. Aspirin has also been found to modulate the expression of key enzymes and proteins involved in metabolic reprogramming in CRC, enhancing the sensitivity of CRC cells to treatment when combined with CB‐839. This suggests that combining Aspirin and CB‐839 could be a promising therapeutic strategy for various tumors [273].

Although targeted therapies hold significant potential for CRC treatment, concerns regarding safety and drug resistance during clinical trials necessitate further investigation (Table 1). High‐throughput technologies will enable comprehensive analyses of therapeutic targets within the genome, epigenome, and metabolome, which could drive the future development of precision and individualized targeted therapies (Table 2).

**TABLE 1:** Clinical trials of targeted therapies for CRC.

Target	Drug	Phase	Trial identifier	Design	References
VEGF	Bevacizumab	Phase III study	NCT00719797	FOLFOXIRI with bevacizumab vs. FOLFOXIRI	[197]
	Ramucirumab	Phase II study	NCT01079780	Irinotecan and cetuximab with/without ramucirumab vs. irinotecan and cetuximab	[198]
EGFR	Cetuximab	Phase III study	NCT00154102	Cetuximab plus FOLFIRI and FOLFIRI alone	[201]
HER2	Trastuzumab deruxtecan	Phase II study	NCT03384940	Trastuzumab deruxtecan	[205]
	Trastuzumab	Phase II study	NCT03225937	Trastuzumab and lapatinib	[206]
		Phase II study	NCT02091141	Trastuzumab and pertuzumab	[207]
BRAF	Vemurafenib	Phase II study	NCT02693535	Cobimetinib and vemurafenib	[208]
	Encorafenib	Phase II study	NCT05217446	Encorafenib, cetuximab, and pembrolizumab	[209]
DNMT	SGI‐110	Phase II study	NCT01896856	Guadecitabine and irinotecan vs. regorafenib or TAS‐102	[5]
GLS	CB‐839	Phase II study	NCT02861300	CB‐839 and capecitabine	[272]

Abbreviations BRAF, B‐raf proto‐oncogene, serine/threonine kinase; FOLFOXIRI, folinic acid, fluorouracil, oxaliplatin and irinotecan; VEGF, vascular endothelial growth factor.

**TABLE 2:** Preclinical trials of targeted therapies for CRC.

Target	Drug	Pathways and mechanisms	Effectiveness	References
HDAC	Vorinostat (SAHA)	Inhibition of the Nrf2–Keap1 pathway	Inhibition of cell proliferation, induction of apoptosis, and cell cycle arrest and upregulation of amelioration of oxaliplatin resistance in human CRC cells	[214, 215]
	Scriptaid	Upregulation of p14 and GADD45G expression levels and downregulation of cyclin B2 and cyclin E2 expression levels	Inhibition of growth and metastasis and induction of cell cycle arrest in CRC	[215]
	Romidepsin (FK228)	Increased acetylation levels of histone H3 and H4 and upregulation of PD‐L1 expression	Inhibition of tumor growth and regulates immune response in vivo	[217]
	Apicidin	Induction of p21WAFI/Cipl and gelsolin	Inhibition of CRC proliferation	[218]
	Chidamide	Inhibition of PI3K/Akt and MAPK/Ras signaling pathways	Induction of apoptosis	[220]
	Valproic acid	Suppression of Wnt signals	Inhibition of CRC proliferation	[223]
GLUT	Phloretin	Activation of the p53 pathway and inhibition of glucose uptake	Inhibition of CRC malignant progression	[81]
HK	2‐DG	Inhibits glycolytic processes	Induction of cell cycle arrest and cell death	[228]
	Metformin	Downregulates PKM2 expression and upregulates IDH1 expression to inhibit the glycolytic process	Slowing the growth of CRC	[230]
PDK	Dichloroacetate (DCA)	Promotes aerobic oxidation and regulates miR‐543/PTEN/Akt/mTOR pathway	Restoration of CRC cell sensitivity to oxaliplatin	[232]
LDH	Oxamate	Inhibits glucose metabolism process and induces mitochondrial apoptosis	Improvement of survival and treatment outcomes when combines with Oxamate	[233]
FASN	Cerulenin	Inhibition of DNA replication and S‐phase cell cycle progression, activation of caspase pathway, inhibition of mTOR signaling pathway	Inhibition of LoVo cell proliferation and induction of apoptosis in CRC cell lines	[240, 241, 242]
	Orlistat	Inhibition of fatty acid synthesis and activation of caspase‐3 pathway	Induction of HT‐29 cell line cell cycle arrest in G1 phase and inhibits proliferation	[249]
	TVB‐3166	Disrupts the structure of lipid rafts, inhibits lipid synthesis, PI3K–AKT–mTOR, β‐catenin signaling pathway, and inhibits the expression of c‐Myc and other oncogenic effectors	Inhibition of xenograft tumor growth	[252]
	Luteolin	Blocking the cell cycle and downregulating the expression level of antiapoptotic proteins	Induction of cell cycle arrest and promotion of apoptosis in HT‐29 human colon cancer cell line	[253]
	Resveratrol	Inhibition of IGF‐1R/Akt/Wnt signaling pathway and activation of p53 protein	Inhibition of CRC proliferation and induces apoptosis	[256]
ACLY	Bempedoic acid (ETC‐1002)	Activates the AMPK pathway thereby inhibiting fatty acid and cholesterol synthesis	IGF1‐mediated HOXA13 overexpression promotes CRC metastasis through upregulating ACLY and IGF1R. Combination treatment of ETC‐1002 with the IGF1R inhibitor linsitinib significantly inhibits HOXA13‐mediated CRC metastasis	[257]
SCD	T‐3764518	Increase in PARP1, a marker of apoptosis inhibited the conversion of stearoyl‐CoA to oleoyl‐CoA	Inhibition of growth and induction of apoptosis in CRC HCT‐116 cells in a CRC xenograft model	[259]
	Betulinic acid	Cell cycle arrest in G2/M phase induces clonogenic incapacity and death of CSCs	Inhibition of CRC proliferation	[260]
CPT1	Etomoxir	Inhibits fatty acid uptake and ATP synthesis	Enhanced apoptotic effect of cisplatin on HCT116 colorectal cells	[264]
	Perhexiline	Increases reactive oxygen species production and apoptosis	Inhibiting gastrointestinal tumor progression	[266]

Abbreviations: c‐Myc, cellular myelocytomatosis proto‐oncogene protein; CSC, cancer stem cell.; HOXA13, homeobox A13; IGF1, insulin‐like growth factor 1; IGF‐1R, insulin‐like growth factor 1 receptor; Nrf2–Keap1, nuclear factor erythroid 2‐related factor 2–Kelch‐like ECH‐associated protein 1; PARP1, poly ADP‐ribose polymerase; PKM2, pyruvate kinase M2; PTEN, phosphatase and tensin homolog.

## 7 Summary and Outlook

CRC ranks as the third most prevalent malignant neoplasm globally, posing substantial challenges in terms of morbidity and mortality. Over the past two decades, research into the pathogenesis of CRC has revealed that genomic alterations accumulated during carcinogenesis are crucial to disease progression. An increasing body of evidence highlights the critical role of specific epigenetic modifications that affect transcriptional mechanisms, thereby influencing gene expression and cellular behavior in CRC. Both genetic and epigenetic alterations impact oncogenes, tumor suppressor genes, and associated signaling pathways, collectively driving the malignant transformation of CRC.

Moreover, alterations in oncogenes and tumor suppressor genes may significantly contribute to the metabolic reprogramming observed in tumor cells. The interaction between cellular metabolism and the epigenome enables CRC cells to adopt more advantageous metabolic pathways, wherein metabolites serve as intermediaries that facilitate epigenetic remodeling and modulate gene expression. Current clinical studies are investigating the development of drugs that target key genes within metabolic and oncogenic pathways, demonstrating promising therapeutic potential.

In conclusion, this review highlights the pathogenesis and metabolic alterations linked to CRC. Addressing specific genetic and metabolic vulnerabilities holds potential for the development of novel therapeutic and diagnostic strategies. Although various inhibitors targeting potential pathways demonstrate promising anticancer effects, the majority are still in preclinical stages. This underscores the necessity for more extensive prospective trials to confirm their clinical efficacy.

The identification of novel tumor‐associated markers and the development of targeted therapies tailored to the diverse needs of patients are of paramount importance. Moreover, integrating targeted therapies with standard treatments or immunotherapies holds the potential to enhance therapeutic efficacy while minimizing toxic side effects, thereby positioning combination therapy as a promising new treatment modality. Additionally, investigating strategies to rectify epigenetic defects arising from metabolite accumulation is warranted. Future research should prioritize elucidating the mechanisms underlying the interplay between epigenetic and metabolic processes, given the complexities of the tumor immune microenvironment and the heterogeneity of CRC, which pose significant challenges to the advancement of targeted therapies. Establishing comprehensive metabolomic and epigenomic analytical methods will be crucial for developing more effective therapeutic strategies for CRC patients.

## Author Contributions

**Yanqi Dang**: design and supervision; revising of original draft. **Guang Ji**: design and supervision; manuscript editing and revision. **Jingyuan Li and Jiashu Pan**: writing original draft. **Lisheng Wang**: manuscript editing and revision. The final manuscript has been reviewed and approved by all the authors.

## Ethics Statement

The authors have nothing to report.

## Conflicts of Interest

The authors declare no conflicts of interest.

## Data Availability

The authors have nothing to report.
